# Interleukin (IL)-18 Binding Protein Deficiency Disrupts Natural Killer Cell Maturation and Diminishes Circulating IL-18

**DOI:** 10.3389/fimmu.2017.01020

**Published:** 2017-08-28

**Authors:** Robert Z. Harms, Austin J. Creer, Kristina M. Lorenzo-Arteaga, Katie R. Ostlund, Nora E. Sarvetnick

**Affiliations:** ^1^Department of Surgery-Transplant, University of Nebraska Medical Center, Omaha, NE, United States; ^2^Mary and Dick Holland Regenerative Medicine Program, University of Nebraska Medical Center, Omaha, NE, United States

**Keywords:** cytokines, inflammation, spleen, mouse models, innate immunity, natural killer cells, interleukin-18, interleukin-18 binding protein

## Abstract

The cytokine interleukin (IL)-18 is a crucial amplifier of natural killer (NK) cell function. IL-18 signaling is regulated by the inhibitory effects of IL-18 binding protein (IL-18BP). Using mice deficient in IL-18BP (IL-18BPKO), we investigated the impact of mismanaged IL-18 signaling on NK cells. We found an overall reduced abundance of splenic NK cells in the absence of IL-18BP. Closer examination of NK cell subsets in spleen and bone marrow using CD27 and CD11b expression revealed that immature NK cells were increased in abundance, while the mature population of NK cells was reduced. Also, NK cells were polarized to greater production of TNF-α, while dedicated IFN-γ producers were reduced. A novel subset of IL-18 receptor α^−^ NK cells contributed to the expansion of immature NK cells in IL-18BPKO mice. Splenocytes cultured with IL-18 resulted in alterations similar to those observed in IL-18BP deficiency. NK cell changes were associated with significantly reduced levels of circulating plasma IL-18. However, IL-18BPKO mice exhibited normal weight gain and responded to LPS challenge with a >10-fold increase in IFN-γ compared to wild type. Finally, we identified that the source of splenic IL-18BP was among dendritic cells/macrophage localized to the T cell-rich regions of the spleen. Our results demonstrate that IL-18BP is required for normal NK cell abundance and function and also contributes to maintaining steady-state levels of circulating IL-18. Thus, IL-18BP appears to have functions suggestive of a carrier protein, not just an inhibitor.

## Introduction

Cytokines are capable of heavily modulating the function and phenotype of immune cells. In the case of natural killer (NK) cells, several cytokines have been shown to induce a profound impact on their attributes [reviewed in Ref. (1)]. Of these, interleukin-18 (IL-18, interferon-γ-inducing factor) is unique, in part, due to its ubiquitous presence in circulation in health and disease [reviewed in Ref. (2)]. Identified over 25 years ago (3), IL-18 functions by signaling through the IL-18 receptor (IL-18R), a functional heterodimer comprised of alpha and beta subunits (4–7). Following ligand binding to the receptor, signaling follows a MyD88-dependent route ultimately capable of activating JNK, p38, and NFkB pathways (8–10). Among NK cells, this signaling can augment proliferation (11), heighten cytotoxicity (12, 13), and contribute to the production of IFN-γ (14, 15).

The management of cytokine signaling requires a diverse range of methods to balance stimulation for proper homeostasis and acute responses. With IL-18, one manner by which signaling is modulated is by the presence of the IL-18 binding protein (IL-18BP). This soluble inhibitor is systemically present in molar excess and has an extremely high affinity for its target (16–18). It is presumed that excessive concentrations of IL-18BP limit the untoward effects of unmitigated IL-18, either by postponing signaling until appropriate conditions are met or by the eventual degradation or elimination of the protein complex. In multiple states of disease, such as schizophrenia, sepsis, viral infection, and autoimmunity (19–22), elevated IL-18 levels are found with IL-18BP concentrations that have ballooned substantially higher than those found in steady-state ratios with IL-18. A similar outcome can be induced clinically, as direct administration of rhIL-18 is followed by heightened levels of circulating IL-18BP (23). The elevation of IL-18BP in response to increased IL-18 is thought to operate *via* IFN-γ signaling, as IFN-γ, a key factor resulting from IL-18 signaling, induces IL-18BP production (24). This feedback loop lessens the potential damage resulting from excessive “free” IL-18 signaling.

The role IL-18BP plays in reducing inflammation is being revealed. For example, the administration of IL-18BP was found to substantially reduce pathology in murine models of experimental arthritis, colitis, endotoxic shock, and type 1 diabetes (25–28). Furthermore, transgenic mice overexpressing IL-18BP are protected from ischemia reperfusion injury (29). Such studies indicate that IL-18BP therapy could be clinically valuable in situations where excessive IL-18 signaling appears to drive disease or enhance its severity. To this end, the therapeutic potential of IL-18BP is being investigated in a current clinical trial for treatment of Adult-onset Still’s disease (https://Clinicaltrials.gov Identifier NCT02398435), an inflammatory disease associated with high plasma levels of IL-18 (30). Yet while the experimental outcomes of augmented IL-18BP levels have received some attention, the consequences of deficiencies in IL-18BP are comparatively much less understood. One recent report demonstrated exacerbated colitis and arrested maturation of goblet cells in the absence of IL-18BP (31). To our knowledge, there have been no further reports nor any indication if IL-18BP deficiency impacts immune cells.

Armed with a diverse array of inhibitory and activating receptors, as well as potent cytotoxic granules and soluble mediators, NK cells are key responders in anti-viral and antitumor immunity [reviewed in Ref. (32)]. Since the functions of NK cells are finely tuned by their cytokine milieu, a detailed assessment of how such factors regulate NK cell function is fundamental in the overall evaluation of NK cell capacities during an immune response. For this purpose, targeted genetic knockout mice provide an avenue for the dissection of molecular function. The importance of IL-18 signaling among NK cells has been shown in IL-18KO or IL-18RαKO mice, with reduced NK cells responses among both genotypes (33, 34). However, the outcome of IL-18BP deficiency on NK cell responses has yet to be investigated. It is thought that early during an immune response, macrophage and/or dendritic cells (DCs) supply NK cells with IL-18 to direct them toward activation and cytokine secretion [reviewed in Ref. (35, 36)]. Thus, it is likely that NK cells without the inhibition of IL-18BP could be abnormally polarized, either from improper cell-to-cell communication or due to freely available IL-18 in circulation. To investigate this, we analyzed splenic and bone marrow NK cells from IL-18BPKO mice using flow cytometry to gauge differentiation state. We observed disrupted maturation and functional polarization among IL-18BPKO NK cells. In querying what was driving these NK cell changes, we found that circulating levels of IL-18 were profoundly diminished in the absence of IL-18BP, yet IL-18 signaling appeared intact and unmitigated.

## Materials and Methods

### Mice

All work described herein was approved by the Institutional Animal Care and Use Committee at University of Nebraska Medical Center (UNMC). Il18bp^tm1(KOMP)Vlcg^ (IL-18BPKO, KOMP repository), Il18^tm1Aki^ (IL-18KO, Jackson), C57BL/6J [(IL-18KO controls) Jackson], and C57BL/6Tac [(IL-18BPKO controls) Taconic] mice used in these studies were derived from breeding colonies at theUNMC. IL-18BPKO mice were generated by a deletion of 1,573 bp starting at position 102,017,311 and ending at position 102,015,739 on chromosome 7. This deletion would effectively knockout known mouse IL-18BP isoforms c and d (17). Further information on the generation of the IL-18BPKO can be found here: http://velocigene.com/komp/detail/12770.

### Flow Cytometry

Single cell suspensions from whole spleens were created by cutting splenic tissue into small pieces and passing through 70 µm nylon screens in RPMI 1640 (HyClone) with 10% FBS (HyClone). Red blood cells were then lysed using ammonium chloride lysis buffer. Bone marrow was isolated from femurs by cleaving bone ends and using a 22 g needle with syringe to flush RPMI 1640 with 10% FBS through the bone. Marrow was disassociated by pipetting and red blood cells were lysed using ammonium chloride lysis buffer. Isolated cells were counted using a hemocytometer and resuspended in PBS, then labeled with Live/Dead Fixable Blue reagent (ThermoFisher) according manufacturer’s recommendation. Cells were then washed with PBS followed by flow cytometry staining buffer (FCSB: PBS with 0.75% BSA, 1 mM EDTA, and 0.05%NaN_3_). Fc receptors were blocked using mixed rat and mouse IgG (ChromePure, Jackson Immunoresearch) followed by incubation with cocktails for antibodies targeting surface proteins for 30 min at 4°C. Cells were washed with FCSB, and when, appropriate, incubated with fluorochrome-conjugated streptavidin for 20 min at 4°C. This was followed by additional washes with FCSB, and cells were then fixed with 3% PFA for 30 min at room temperature. Following fixation, we washed cells twice before resuspension in FSCB prior to analysis. Cells were analyzed using a BD LSR II within 24 h of labeling and fixation.

For intracellular flow cytometry experiments, isolated splenocytes were cultured with RPMI 1640 (Hyclone) with 10% FBS (Hyclone), 2.05 mM l-glutamine (HyClone), 50 I.U./mL penicillin, 50 µg/mL streptomycin (Corning), and 50 µM β mercaptoethanol (Sigma) and stimulated with either 50 ng/mL IL-18 (MBL), 2 ng/mL IL-2 (Cell Sciences), both IL-18 and IL-2, 500 ng/mL LPS *Escherichia coli* 026:B6 (Sigma Aldrich), or nothing. Following overnight culture, splenocytes were treated with Cell Stimulation Cocktail (a mixture of phorbol 12-myristate 13-acetate (PMA), ionomycin, brefeldin A, and monensin, eBioscience) or brefeldin A and monensin (BioLegend) as control for 5 h. Splenocytes were surface labeled as described above. Following fixation and wash, cells were washed twice with permeabilization buffer (BioLegend) and then resuspended in permeabilization buffer and Fc receptors were blocked again as above. Permeabilized cells were then incubated with cocktails of antibodies for 30 min at 4°C, washed with permeabilization buffer, then washed with FCSB before final resuspension prior to analysis as described above. Flow cytometry data were analyzed using FlowJo (Treestar).

The following monoclonal antibodies and reagents were used: CD27 (LG.3A10, BV421 Biolegend); CD19 (6D5, Biotin BioLegend); Ly-6G (1A8, Biotin BioLegend); CD3 (17 A2, Biotin and FITC BioLegend); CD45 (30-F11, BUV395 BD Biosciences); TER-119 (TER-119, Biotin BioLegend); CD11b (M1/70, PerCP-Cy5.5 Biolegend and BV605 BD Biosciences); KLRG1 (2F1, PE BioLegend); NK1.1 (PK136, PE-Cy7 BioLegend); NKp46 (29A1.4, PE and APC BioLegend); CD122 (TM-β1, PE BioLegend); NKG2A/C/E (20d5 FITC eBioscience); Ly-49D (4E5 FITC BioLegend); Ly-49C/I (5E6, FITC BioLegend); Ly-49H (3D10, FITC BioLegend); Ly-49G2 (eBio4D11, FITC eBioscience); CD244.2/2B4 (eBio244F4, PE eBioscience); CD314/NKG2D (CX5, PE eBioscience); CD49b (DX5, PE BioLegend); TNF-α (MP6-XT22, PE BioLegend); IFN-γ (XMG1.2, eFluor450 eBioscience); IL-18Rα (112614, APC R&D Systems); streptavidin [BUV395 (BD Biosciences); and APC-Cy7 (BioLegend)]. Although not used to generate the data shown on IL-18Rα expression among NK cells, we observed extremely similar results during panel optimization when we compared IL-18Rα clone 112614 (R&D) with IL-18Rα clone BG/IL18RA from BioLegend.

### Western Blotting

Single cell suspensions from whole spleens from seven IL-18BPKO and eight C57BL/6Tac female mice age 16–20 weeks were created as described above. To generate cytosolic lysates of the splenocyte preparations, we used M-PER Mammalian Protein Extraction Reagent (ThermoScientific) with the addition of PMSF, Protease Inhibitor Cocktail III, and Phosphatase Inhibitor II (Research Products International). Following lysis, samples were spun at 21,000 × *g* for 10 min to pellet debris and protein concentration was calculated using Protein Assay Dye Reagent (BioRad) and then diluted into Laemmli buffer. Samples were heated to 95°C for 5 min, then 20 µg of lysate per lane of was loaded onto 15% polyacrylamide gels, separated, and then transferred onto nitrocellulose. Murine recombinant IL-18 (MBL) was used at 1 ng per lane as a positive control. Blots were blocked with 5% non-fat milk, then probed with IL-18 (1:1,000, Abcam, rabbit polyclonal ab71495). HRP-conjugated donkey anti-rabbit IgG (Jackson Immunoresearch) was used at 1:20,000 and Clarity Western ECL Substrate (BioRad) was used prior to exposure. Blots were then stripped and reprobed with β tubulin antibodies (SantaCruz, rabbit polyclonal, H-235). Secondary antibody and ECL were used as for IL-18. IL-18 antibody specificity was confirmed using IL-18KO splenic lysates. While non-specific bands were present at ~32 kD, ~70 kD, and ~125 kD, a specific band of ~23 kD fitting with pro-form IL-18 was observed in wild-type (WT) splenic lysates but not among IL-18KO lysates (Figure S1 in Supplementary Material). Surprisingly, an alternate antibody for IL-18 (Santa Cruz, goat polyclonal sc-6179) produced non-specific bands in WT and IL-18KO splenic lysates at ~22 kD, which could easily be misconstrued for pro-form IL-18 (data not shown).

### Immunohistochemistry (IHC) and Immunofluorescence (IF)

Whole spleens from IL-18BPKO, IL-18KO, and C57BL/6Tac mice were fixed in 10% neutral buffered formalin for 48 h prior to embedding with paraffin. All sections were cut to 4 µm thickness. Sections were then deparaffinized with xylene and rehydrated with gradated ethanol. For antigen retrieval, we used the 2100 Antigen Retriever (Aptum Biologics, Ltd.) with R-universal epitope recovery buffer (Electron Microscopy Sciences) according to manufacturer’s recommendation. During antibody validation and titration, we observed that for both IL-18 (Abcam, rabbit polyclonal ab71495) and IL-18BP (Santa Cruz, goat polyclonal S-19) detection, R buffer was superior to 10 mM sodium citrate for antigen retrieval (data not shown). Titration results demonstrated that ideal staining for IL-18 was at 2 µg/mL and IL-18BP was at 6 µg/mL (data not shown). Interestingly, four other polyclonal antibodies [IL-18BPd (AF122) and IL-18BPc (AF129, both R&D); IL-18BP (141012, US Biological Life Sciences); IL-18BP (Santa Cruz, H-61)] were tested for IL-18BP specificity but none produced specific staining histologically (data not shown).

Following antigen retrieval, we blocked endogenous peroxidases and phosphatases using Bloxall (Vector Labs), and blocked potential endogenous biotin with the Avidin/Biotin Blocking System (BioLegend). Sections were then blocked with Section Block (Electron Microscopy Sciences) to reduce non-specific binding of antibody.

For IHC, primary antibodies were diluted into 5% normal donkey serum (Equitech-Bio, Inc.) at the concentrations given above and applied to sections for 1 h at room temperature. After washing, we applied biotinylated donkey anti-goat IgG and biotinylated donkey anti-rabbit IgG (Jackson Immunoresearch). We then utilized the ABC Elite kit (Vector Labs) and visualized antibody localization using DAB Quanto (Thermo Scientific), both according to manufacturers’ recommendations. Finally, sections were counterstained with haemotoxylin, dehydrated and mounted with Cytoseal XYL (Thermo Scientific). Whole slides were scanned with a Coreo Au Slide Scanner (Ventana) and images from scanned slides were taken using the Ventana image viewer.

For IF, TrueBlack Lipofuscin Autofluorescence Quencher (Biotium) was used according to manufacturer’s recommendation to reduce tissue autofluorescence prior to the application of Section Block. Primary antibodies were applied in a 2-step process. IL-18BP was applied first as above. To amplify IL-18BP signal, we targeted the IL-18BP with chicken anti-goat IgG (Abcam), followed by AF488-conjugated donkey anti-chicken IgY (Jackson ImmunoResearch). Next, IL-18 (described above), Mac-3 (BD Biosciences, rat monoclonal M3/84, 1:100), and CD3 (Abcam, rabbit polyclonal ab5690, 1:100) antibodies were applied for 1 h at room temperature. Biotinylated donkey anti-rat IgG and anti-rabbit IgG (Jackson ImmunoResearch) and streptavidin-conjugated CF555 (Biotium) at 8 µg/mL were then used for detection. Sections were mounted with ProLong Diamond antifade mountant with DAPI (Molecular Probes). Photos were taken using a Zeiss AxioCam 503 mono camera attached to Zeiss Axio Imager.A2 using Zeiss Zen software. We merged and prepared images with ImageJ.

### Intraperitoneal (i.p.) LPS Injection

Mice were administered an i.p. injection of 35 µg LPS from *E. coli* 026:B6 (Sigma Aldrich) diluted into 500 µL saline. This quantity of LPS is in the range of the protective amount [see Ref. (37)]. After 8 h, submandibular blood and tissues were collected from each animal. Blood was collected using Goldenrod lancets (Medipoint, Inc.) in K_2_ EDTA microtainers (BD Biosciences) and centrifuged at 10,000 × *g* for 5 min. Plasma was removed, aliquoted, and stored at −80°C until use. ELISAs for IL-18 (eBioscience) and IFN-γ (BioLegend) were performed according to manufacturers’ recommendations.

### Thioglycollate-Elicited Peritoneal Macrophage (TEM)

Female C57BL/6Tac and IL-18BPKO mice were injected i.p. with 1.5 mL 3% thioglycollate (Becton Dickinson). Four days post injection, animals were euthanized and their peritoneal cavities were flushed with 10 mL PBS twice. Recovered peritoneal exudate cells were pooled from three mice per genotype per experiment. Peritoneal exudate cells were washed two times with PBS, resuspended in complete RPMI 1640 (HyClone) containing 10% FBS (HyClone), 2.05 mM l-glutamine (HyClone), 50 I.U./mL penicillin, 50 µg/mL streptomycin (Corning), and 25 mM HEPES (HyClone), and then plated at 2 × 10^6^ per well in 12 well plates. Three hour post-plating, plates were washed two times with complete RPMI to remove non-adherent cells, thereby enriching for macrophage. Adherent peritoneal macrophage was then cultured with complete RPMI (control) or complete RPMI with 1 µg/mL LPS from *E. coli* 026:B6 (Sigma Aldrich). After 24 h, the supernatants were removed, centrifuged, aliquoted, and stored at −80°C until use. Plates were washed two times with complete RPMI to remove any residual cytokines, and then treated with 1 mL complete RPMI (control) or 1 mL complete RPMI with nigericin (Tocris) at varying molarity for 90 min. These supernatents were then removed, centrifuged, aliquoted, and stored at −80°C until analysis. ELISAs for IL-18 (eBioscience), IL-1β, and IL-6 (BioLegend) were performed according to manufacturers’ recommendations.

### Statistics

We performed statistical analysis and constructed figures with GraphPad Prism v6.03 (GraphPad Software, Inc.). Significance was determined using the following non-parametric tests: Mann–Whitney *U* test for comparisons of two groups, and Kruskal–Wallis test for comparison of three groups. *p*-Values less than 0.05 are considered to be statistically significant. Further information, including sample sizes and number of replicates is provided in the legends accompanying each figure. For Table 1, fold change was calculated using mean proportion following stimulation as dividend and mean proportion of freshly isolated NK cells as divisor. The resulting quotient was subtracted by 1. Thus, for example, a fold change of 0.8 represents an 80% increase from baseline.

## Results

### IL-18BPKO Mice Harbor Reduced Numbers of Splenic CD27^−^, CD11b^+^ NK Cells and Increased Numbers of CD27^+^, CD11b^−^ NK Cells

**Figure 1:**
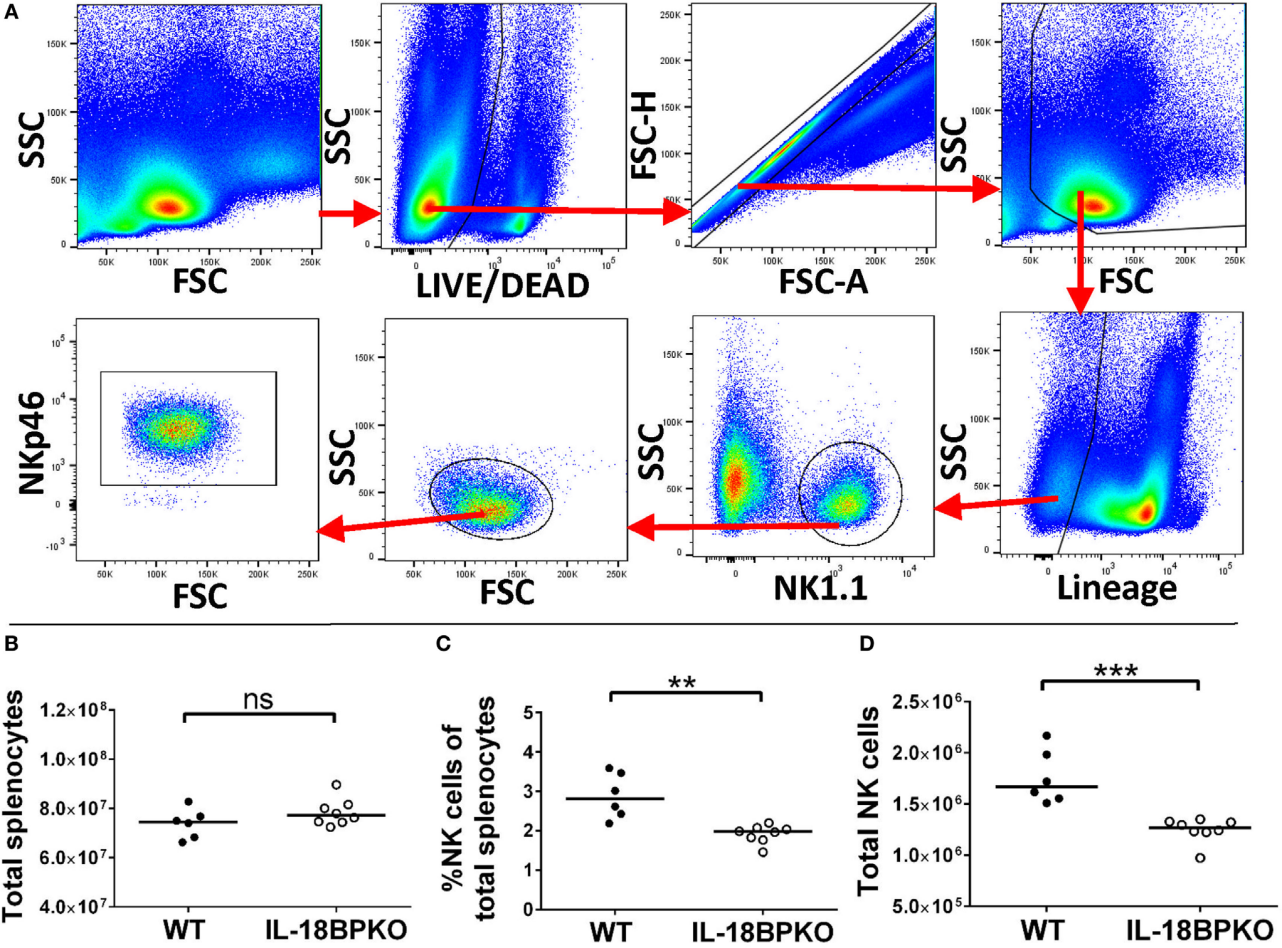
Splenic natural killer (NK) cells are reduced in abundance among interleukin (IL)-18BPKO mice. **(A)** Identification of splenic NK cells. Living, singlet events were selected from total splenocytes. After gating out debris, we selected lineage (CD3, CD19, Ly-6G), NK1.1^+^ events as NK cells. NK cell phenotype was confirmed with NKp46 labeling. Total splenocytes **(B)** were relatively similar between wild-type (WT) and IL-18BPKO mice, yet the proportion **(C)** and absolute number **(D)** of NK cells was significantly reduced among IL-18BPKO mice. Results from three unique experiments from six WT and eight IL-18BPKO mice. Bars represent median. Significance calculated with Mann–Whitney *U* test. **p < 0.01, ***p < 0.001, ns, not significant.

Assuming that NK cells without the inhibition of IL-18BP could be abnormally polarized, we investigated NK cells from IL-18BPKO mice for maturational and functional differences. We identified NK cells using the gating strategy shown in Figure 1A. In brief, NK cells were defined as NK1.1^+^, lineage (CD3, CD19, and Ly-6G), living, singlet splenocytes. Cross-confirmation using anti-NKp46 antibodies revealed this approach successfully identified NK cells. Although splenocyte numbers were similar among IL-18BPKO and WT mice (Figure 1B), we observed both a decrease in proportion and number of NK cells among IL-18BPKO mice compared to WT (Figures 1C,D). Thus, it immediately became apparent that IL-18BP influences NK cell homeostasis.

Natural killer cell development can be delimited using the relative surface expression of CD27, CD11b, and killer cell lectin such as receptor G1 (KLRG1). The TNF receptor family member CD27 is expressed by immature NK cells, which have been shown to highly proliferative and be less cytotoxic than those lacking CD27 (38). CD11b (or integrin alpha-M) functions as a cellular adhesion molecule capable of binding ICAM or as a complement receptor for iC3b [reviewed in Ref. (39)]. CD11b expression increases with NK cell maturation and has been demonstrated to denote enhanced effector functions (40, 41). Finally, KLRG1 is an inhibitory receptor with cadherin binding capability [see Ref. (42)]. Expression of KLRG1 by NK cells denotes lower proliferation and turnover than among KLRG1^−^ NK cells (43).

**Figure 2:**
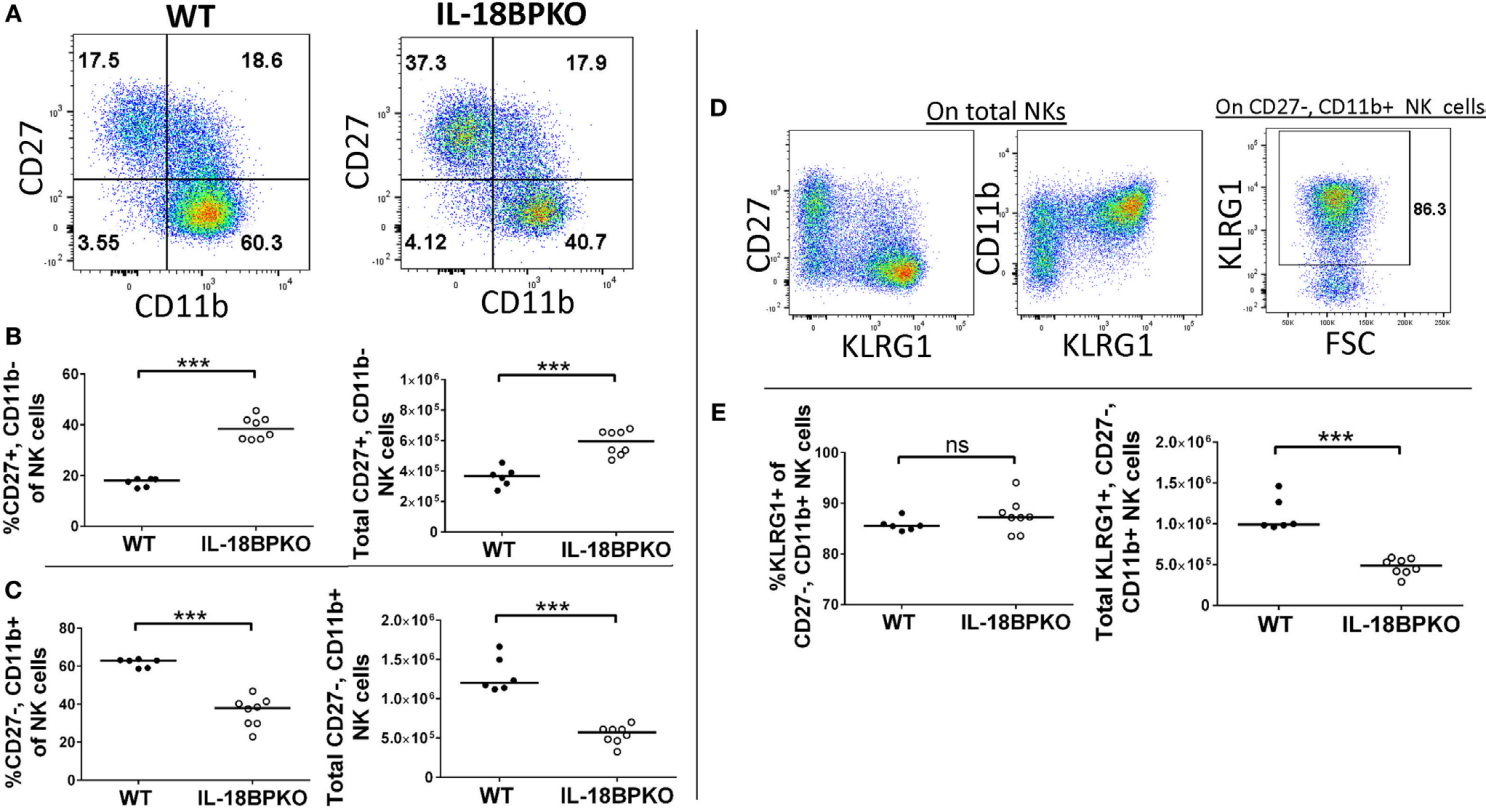
In the absence of interleukin-18 binding protein (IL-18BP), CD27^+^, CD11b^−^ natural killer (NK) cells are increased in abundance while CD27^−^, CD11b^+^ NK cells, including terminally differentiated KLRG1-expressors, are reduced in abundance. **(A)** Representative gating of CD27 versus CD11b on wild type (WT) and IL-18BPKO splenic NK cells. **(B)** Immature CD27^+^, CD11b^−^ NK cells are increased in proportion and abundance in the spleens of IL-18BPKO mice, while **(C)** mature CD27^−^, CD11b^+^ NK cells are reduced in proportion and abundance. **(D)** Representative dot plots showing KLRG1 expression versus either CD27 or CD11b and KLRG1 gating on CD27^−^, CD11b^+^ NK cells. KLRG1 expression among NK cells is mostly among the CD27^−^, CD11b^+^ compartment. **(E)** Although the proportion of KLRG1 expressers within the CD27^−^, CD11b^+^ compartment was relatively similar between the two genotypes, the overall abundance of KLRG1^+^, CD27^−^, and CD11b^+^ was reduced among IL-18BPKO mice. Results from three unique experiments from six WT and eight IL-18BPKO mice. Bars represent median. Significance calculated with Mann–Whitney *U* test. ***p < 0.001, ns, not significant.

Following this model of NK cell differentiation, we divided NK cells according to CD27, CD11b, and KLRG1 expression to determine if IL-18BP influences NK cell differentiation. We observed striking differences in NK cell maturation between the two genotypes (Figures 2A–C). Immature CD27^+^, CD11b^−^ NK cells were expanded in proportion and number among IL-18BPKO mice compared to controls (Figure 2B), while CD27^+^, CD11b^+^ NK cells were relatively similar between the IL-18BPKO mice (data not shown). Interestingly, CD27^−^, CD11b^+^ NK cells were significantly reduced in proportion and number among IL-18BPKO mice (Figure 2C). There was no difference observed among CD27^−^, CD11b^−^ NK cells (data not shown). Accordingly, KLRG1-expressing NK cells [being chiefly CD27^−^, CD11b^+^ among both WT and IL-18BPKO (Figure 2D)] were also reduced in overall number among IL-18BPKO mice (Figure 2E). These results demonstrate that NK cells in IL-18BPKO mice display disrupted maturation. The population of less mature, highly proliferative CD27^+^, CD11b^−^ NK cells is expanded, while mature CD27^−^, CD11b^+^ NK cells, including KLRG1-expressers, are substantially reduced.

### IL-18BPKO NK Cell Maturation Is Similarly Altered in the Bone Marrow

**Figure 3:**
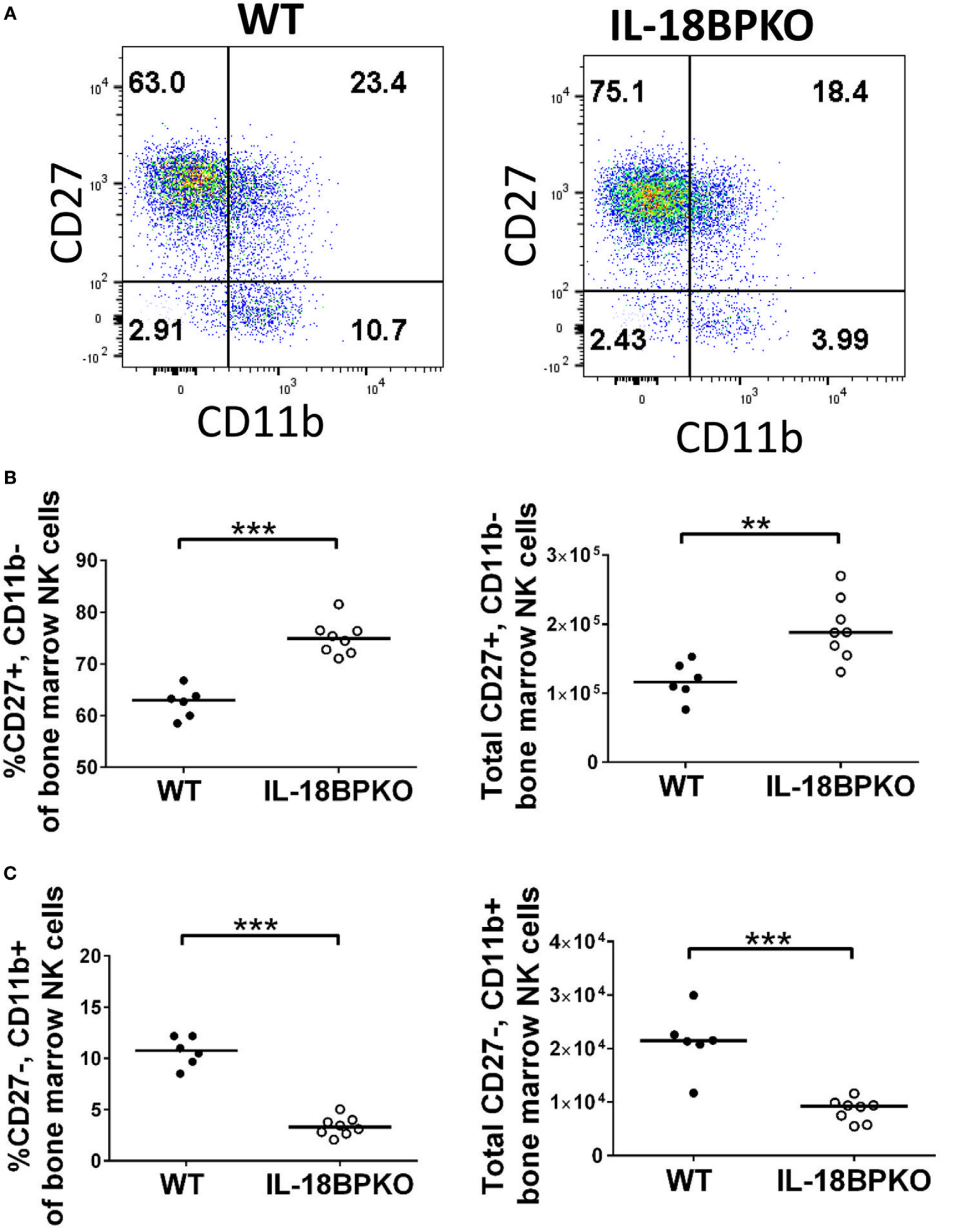
Similar to what we observed in the spleen, CD27^+^, CD11b^−^ bone marrow natural killer (NK) cells are increased in abundance while CD27^−^, CD11b^+^ bone marrow NK cells are reduced in abundance among interleukin (IL)-18BPKO mice. **(A)** Representative gating of CD27 versus CD11b on bone marrow NK cells. **(B)** CD27^+^, CD11b^−^ NK cells are increased in proportion and abundance in the bone marrow of IL-18BPKO mice, while **(C)** CD27^−^, CD11b^+^ NK cells are reduced in proportion and abundance. Results from three unique experiments from six WT and eight IL-18BPKO mice. Bars represent median. Significance calculated with Mann–Whitney *U* test. **p < 0.01, ***p < 0.001.

At the same time as the splenic analysis, we investigated bone marrow for NK cell differences. Gating and identification of bone marrow NK cells are shown in Figure S2A in Supplementary Material. Interestingly, unlike in the spleen, overall bone marrow NK cell proportion was slightly elevated among IL-18BPKO mice and yet numbers of total NK cells were not significantly different (Figures S2B–D in Supplementary Material). However, bone marrow NK cells from IL-18BPKO mice were similarly disrupted as we observed in the spleen (Figures 3A–C). CD27^+^, CD11b^−^ cells were increased in proportion and number while CD27^−^, CD11b^+^ NK cells were reduced (Figures 3B,C). Thus, NK cells in the bone marrow are also impacted by IL-18BP deficiency. In combination, our splenic and bone marrow data reveal that IL-18BP has a profound effect on NK cells. In its absence, immature NK cells are expanded in lymphoid tissue, while mature NK cells are reduced. Furthermore, total splenic NK cell abundance is reduced with presumably unmitigated IL-18 signaling and it may be that the slight elevation of total NK cells that we observed in the bone marrow is to compensate for peripheral reductions.

### NK Cells from IL-18BPKO Mice Are Polarized to Increased TNF-α Production

In an environment with freely accessible IL-18, it is likely that IFN-γ responses could be amplified among NK cells. However, our phenotypic data revealed that normal NK cell maturation is impacted in the absence of IL-18BP. Thus, deranged cytokine output could be expected. To investigate this, we isolated splenocytes from IL-18BPKO and WT mice and stimulated them with IL-18, IL-18 with the prosurvival cytokine IL-2, or indirectly *via* LPS. Following overnight incubation with the stimulants described above, we then treated NK cells with PMA and ionomycin to elicit robust cytokine production. Splenocyte cultures were then labeled for IFN-γ and TNF-α expression and analyzed by flow cytometry.

**Figure 4:**
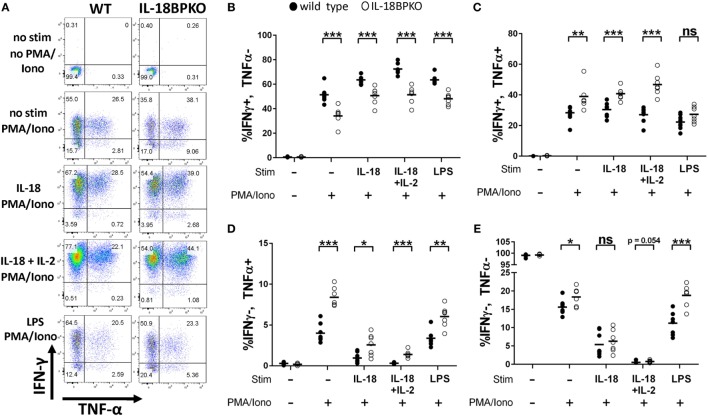
Natural killer (NK) cells from interleukin (IL)-18BPKO mice are polarized toward greater TNF-α production and reduced IFN-γ production. NK cells were stimulated for 24 h with either IL-18 (50 ng/mL), IL-18 (50 ng/mL)^+^ IL-2 (2 ng/mL), or indirectly with LPS (500 ng/mL) and then treated with PMA and ionomycin (PMA/Iono) for 5 h prior to intracellular labeling of IFN-γ and TNF-α. **(A)** Representative gating for each treatment from wild-type (WT) and IL-18BPKO mice. **(B)** The proportion of IFN-γ^+^, TNF-α^−^ NK cells was reduced among unstimulated and stimulated NK cells. **(C,D)** IFN-γ^+^, TNF-α^+^, and IFN-γ^−^, TNF-α^+^ NK cells from IL-18BPKO mice were increased among unstimulated and stimulated NK cells. **(E)** IFN-γ^−^, TNF-α^−^ NK cells were elevated among IL-18BPKO mice in unstimulated and LPS stimulated groups. Results from two unique experiments from eight WT and seven IL-18BPKO mice. Bars represent median. Significance calculated with Mann–Whitney *U* test. *p < 0.05, **p < 0.01, ***p < 0.001, ns, not significant.

Surprisingly, we observed that NK cells from IL-18BPKO were polarized to greater TNF-α production rather than IFN-γ production (Figures 4A–E). Indeed, the proportion of total IFN-γ^+^, TNF-α^−^ IL-18BPKO NK cells was reduced following all stimulations as well as among unstimulated splenocytes (Figure 4B). Alternatively, IFN-γ^+^, TNF-α^+^, and IFN-γ^−^, TNF-α^+^ NK cells were increased among NK cells from IL-18BPKO mice (Figures 4C,D). Interestingly, among unstimulated and indirectly stimulated (LPS) NK cells, the proportion of IFN-γ−, TNF-α^−^ NK cells was also increased among IL-18BPKO mice (Figure 4E), suggesting an expanded, undifferentiated compartment.

The outcomes of these stimulations demonstrate that not only does IL-18BP influence NK cell maturation and abundance, it also directs NK cell effector status. TNF-α, in addition to being a potent agent of cell death, is a stimulatory factor capable of inducing NFκB signaling (44). Thus, the absence of IL-18BP generates NK cells that are uniquely polarized and capable of enhanced TNF-α release that could lead to heightened cytotoxicity or inflammation.

### CD27^+^, CD11b^−^ NK Cells Comprise IL-18Rα^+/−^ Subsets, Both of Which Contribute to the Expansion Observed in IL-18BPKO Mice

In an environment of mismanaged IL-18 signaling brought about by the removal of IL-18BP, immature NK cells are increased while mature NK cells are decreased. This could indicate that IL-18 responsiveness among NK cell subsets is differentially regulated, perhaps by IL-18R expression. The IL-18R is a heterodimer comprised of alpha and beta chains, with IL-18Rα augmenting ligand binding within the dimer, while IL-18Rβ functions in signaling (4–7). Previous reports of murine NK cell surface expression of IL-18R are not in agreement and none analyzed NK cell subsets (11–13). It is plausible that NK cell subsets possess varying levels of IL-18R, which would suggest enhanced or diminished responsiveness to IL-18. Such receptor variance could help elucidate some of the maturational differences we have observed.

**Figure 5:**
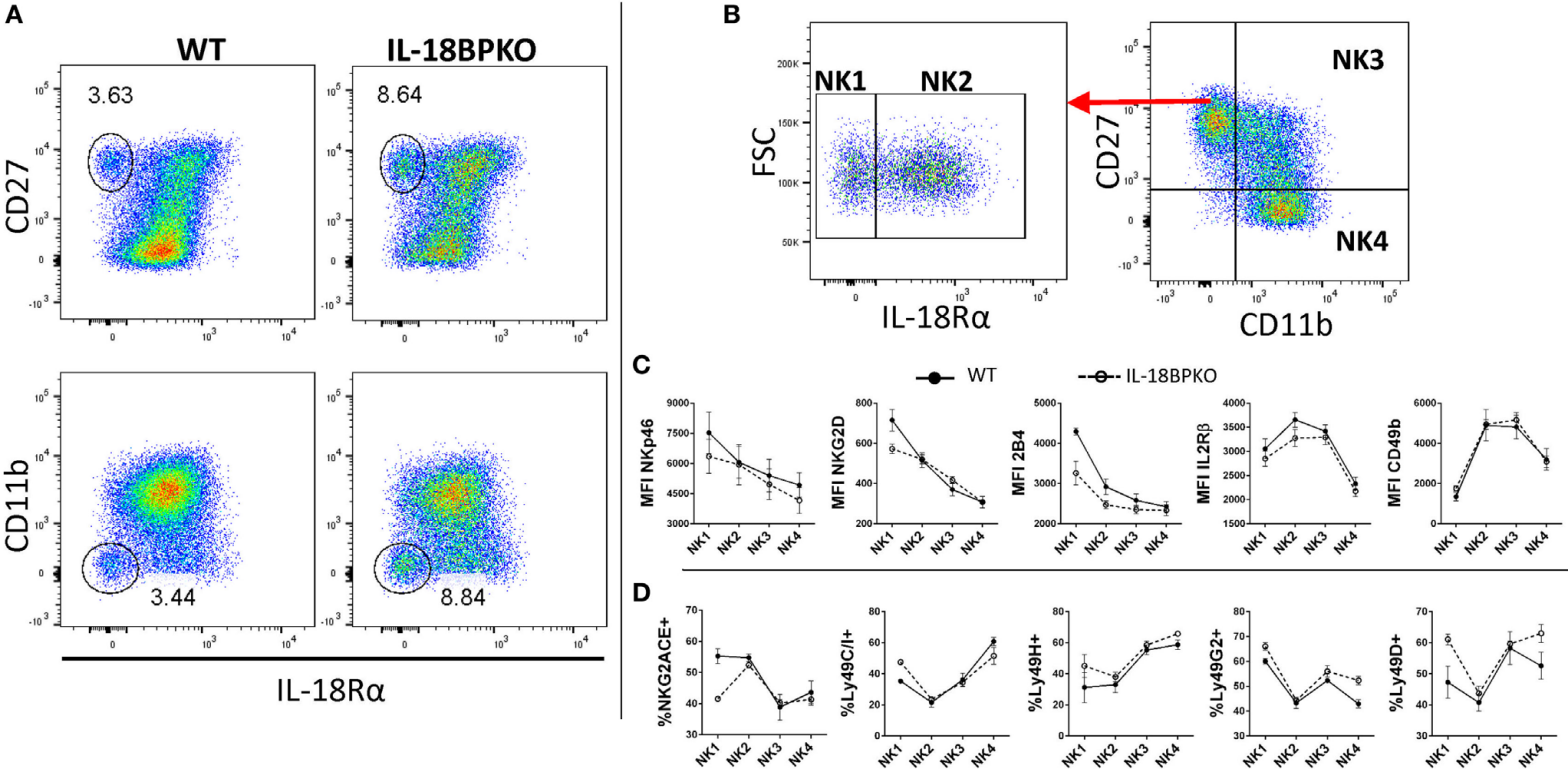
Traditionally defined natural killer (NK) cells harbor an interleukin-18 receptor α^−^ (IL-18Rα^−^) compartment that is CD27^+^, CD11b^−^, which contributes to the expansion observed in IL-18BPKO mice. Phenotypic examination of IL-18Rα^−^ NK cells reveals a full complement of NK cell-associated surface proteins but with an immature CD49b^low^ phenotype. **(A)** We examined NK cells from WT and IL-18BPKO mice for IL-18Rα expression to determine if any NK cell compartment was more sensitive to IL-18 signaling based on receptor abundance. Interestingly, both lines possessed a previously undescribed IL-18Rα^−^ subset. This subset contributes to the proportional expansion of CD27^+^, CD11b^−^ NK cells in IL-18BPKO mice. Note that among IL-18Rα^+^ NK cells, higher IL-18Rα expression was associated with higher CD27 expression. Data shown are representative of four experiments and six animals per genotype. **(B)** NK cells were defined in a maturational continuum according to IL-18Rα, CD27, and CD11b expression as such: IL-18Rα^−^, CD27^+^, CD11b^−^ (NK1); IL-18Rα^+^, CD27^+^, CD11b^−^ (NK2); IL-18Rα^+^, CD27^+^, CD11b^+^ (NK3); and IL-18Ra^+^, CD27^−^, CD11b^+^ (NK4). **(C)** While all subsets were positive for the surface proteins (see Figure S3A in Supplementary Material), NK1 cells had the highest levels of expression for NKp46, NKG2D, and 2B4. Alternatively, CD49b and IL-2Rβ started with lower expression before elevating and plateauing, then decreasing with maturity. **(D)** The proportion expressing NKG2A/C/E and Ly49H, D, G2, and C/I varied somewhat similarly with maturation state. Panels **(C,D)** are results of three separate experiments, one wild type (WT), and one IL-18BPKO per experiment. Circles are mean values and error bars represent SD. Close circles with solid line are WT. Open circles with dashed line are IL-18BPKO.

To investigate if IL-18R expression varies with NK cell maturation, we first examined NK cells from WT and IL-18BPKO mice for expression of IL-18Rα (four experiments, six mice per genotype). Our results confirmed that NK cells are mostly (>95% in WT) IL-18Rα^+^ (Figure 5A), in line with one previous report (11). However, we observed the presence of an IL-18Rα^−^ CD27^+^, CD11b^−^, NK cell subset among WT and IL-18BPKO mice, and these IL-18Rα^−^ NK cells were also proportionally expanded in IL-18BPKO mice (Figure 5A). Importantly, IL-18Rα^−^ NK cells were also observed in the bone marrow as well as among IL-18KO mice and their respective WT controls (data not shown). Once upregulated, IL-18Rα-expression appears greatest among NK cells with the highest CD27 expression and declines slightly with NK cell maturity (Figure 5A). Since the contraction of NK cells observed in the absence of IL-18BP is chiefly among CD27^−^, CD11b^+^ NK cells (where we observe slightly lower IL-18Rα expression), the expression of patterns of IL-18Rα alone cannot fully explain the observed difference in IL-18BPKO mice.

IL-18Rα^−^ NK cells reside within the CD27^+^, CD11b^−^ compartment, which has been shown to harbor immature NK cells that give rise to terminally differentiated subsets (38, 40). We hypothesized that the expression of IL-18Rα occurs during maturation and that the IL-18Rα^−^ NK cell subset is actually antecedent to IL-18Rα^+^ NK cells. A paradigm for NK cell maturation has been established using CD27 and CD11b (40). Therefore, we incorporated IL-18Rα expression into this model. We designated NK cells as IL-18Rα^−^, CD27^+^, CD11b^−^ (NK1); IL-18Rα^+^, CD27^+^, CD11b^−^ (NK2); IL-18Rα^+^, CD27^+^, CD11b^+^ (NK3); and IL-18Rα^+^, CD27^−^, CD11b^+^ (NK4) in a maturational continuum (Figure 5B). We then analyzed these subsets for expression of relevant NK cell surface proteins (three experiments, one IL-18BPKO, and one WT per experiment).

All NK cell subsets were positive for NKp46, 2B4, NKG2D, IL-2Rβ, and CD49b (see Figure S3A in Supplementary Material), confirming an overall NK cell phenotype for this novel IL-18Rα^−^ compartment. Furthermore, our results indicate that the hypothesized NK1 → NK4 maturation follows similar trends among WT and IL-18BPKO mice (Figure 5C) NKp46, NKG2D, and 2B4 had highest expression among NK1 sloping downward with maturation. Alternatively, IL-2Rβ and CD49b were lowest among the NK1 subset before plateauing and decreasing with maturation. Interestingly, high CD49b expression has previously been shown to be late in the maturation of NK cell precursors and following acquisition of NK1.1 (45).

We also examined NK cell subsets for expression of activating and inhibitory receptors of the Ly49 family and NKG2A/C/E (46, 47). For representative gating, see Figure S3B in Supplementary Material. Again, similar maturation trends were observed among WT and IL-18BPKO mice for Ly49C/I, Ly49H, Ly49G2, and Ly49D (Figure 5D). However, we observed that the NK1 subset among IL-18BPKO mice had lower proportions of NKG2A/C/E expressers than WT mice, although what drives this deficiency is unclear. Nevertheless, the combined phenotypic data characterize a previously undescribed NK cell subset lacking IL-18Rα expression. These seemingly immature IL-18Rα^−^ NK cells contribute to (but do not fully account for) the expansion of CD27^+^, CD11b^−^ cells observed in IL-18BPKO mice.

### IL-18BPKO Mice Do Not Have Detectable Levels of Circulating IL-18, but Possess Similar Levels of Pro-Form IL-18 in the Spleen

**Figure 6:**
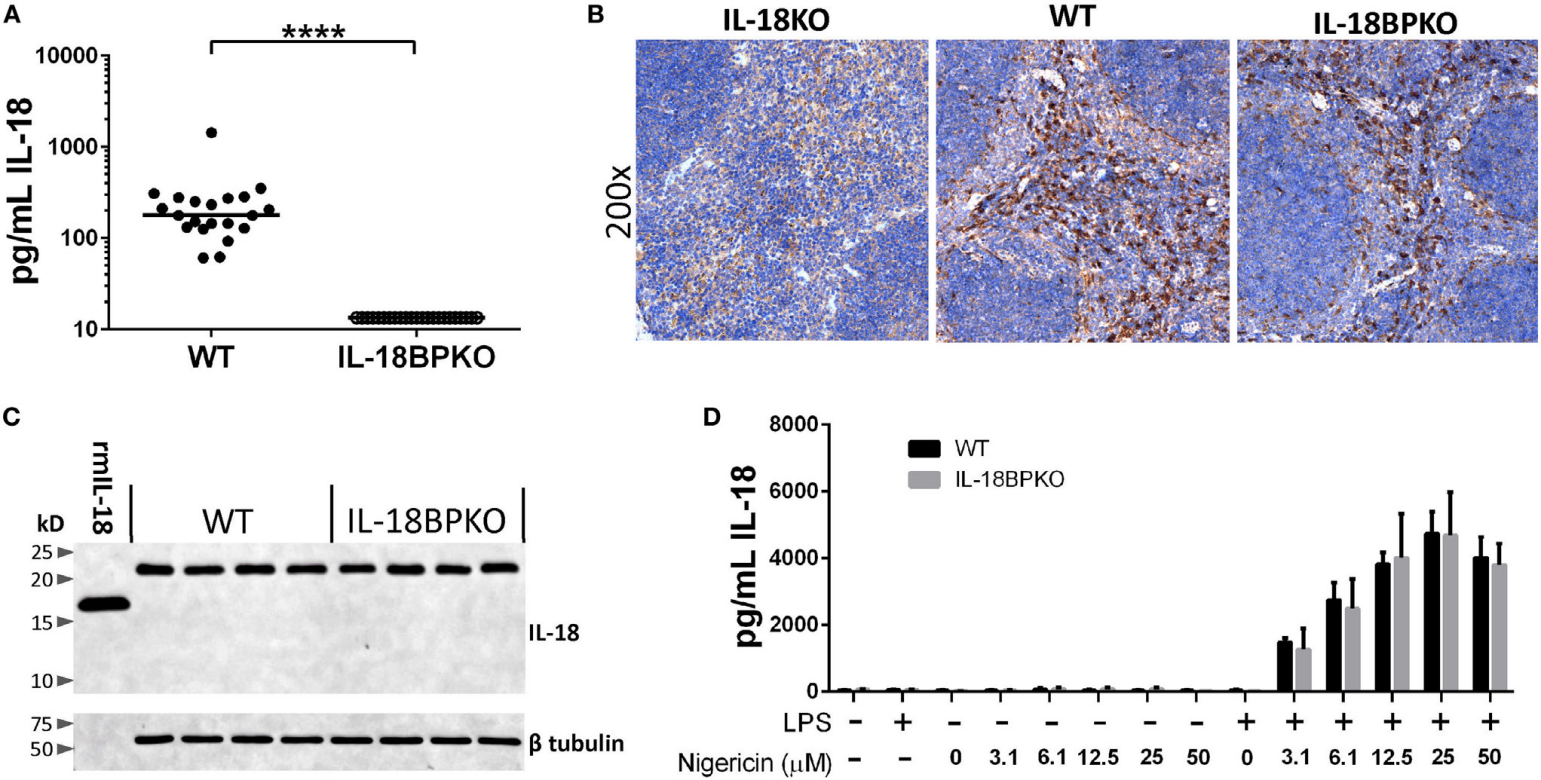
Circulating IL-18 levels are significantly reduced in interleukin (IL)-18BPKO mice, yet they produce comparable amounts of pro-form IL-18 as wild type (WT), and release similar amounts of IL-18 following stimulation. **(A)** IL-18 ELISA results from submandibular plasma from WT (*N* = 21) and IL-18BPKO (*N* = 23) male and female mice. IL-18 levels were below limit of detection among all tested IL-18BPKO animals. Bars represent median. Significance calculated with Mann–Whitney *U* test (****p < 0.0001). **(B)** Representative IL-18 immunohistochemistry results from splenic sections from WT (*N* = 10), IL-18BPKO (*N* = 10), and IL-18KO (control) animals. Splenic IL-18-producing cells are similarly distributed among WT and IL-18BPKO mice and are most abundant within the red pulp, likely among red pulp macrophage. However, IL-18-expressing cells can also be observed in the marginal zone as well as within the white pulp. **(C)** Splenocyte lysates from WT and IL-18BPKO mice contain similar amounts of pro-form IL-18. Western blot showing pro-form IL-18 (~23 kD) is present in both WT and IL-18BPKO splenic lysates. We observed no mature or cleaved forms of IL-18 in lysates from WT and IL-18BPKO mice. 1 ng of recombinant murine IL-18 (rmIL-18) was ran as a control. Representative blot from total of eight WT and seven IL-18BPKO splenic lysates. **(D)** IL-18BPKO thioglycollate-elicited peritoneal macrophage (TEM) release comparable amounts of IL-18 as WT TEM following stimulation. TEM were cultured in the presence or absence of LPS (1 µg/mL) overnight. Cultures were then washed and treated with increasing concentrations of nigericin for 90 min to activate the NLRP3 inflammasome and promote release of mature IL-18. We observed no difference in IL-18 release between WT and IL-18BPKO TEM at any concentration of nigericin. Data are from combined results of three separate experiments per genotype, using pooled TEM from three mice per experiment. Bars represent mean and error bars are SD.

The absence of IL-18BP results in a highly modified NK cell compartment. Presumably, without the inhibition provided by IL-18BP, IL-18 signaling is unabated. It is previously been demonstrated that IL-18 signaling promotes the production of IFN-γ which in turns drives IL-18BP production, thereby providing a negative feedback loop during times of enhanced mature IL-18 release. It is unknown how steady state circulating IL-18 levels may be impacted with the removal of a key component of this signaling dynamic. To answer this question, we measured IL-18 levels among male and female IL-18BPKO (*N* = 23) and WT (*N* = 21) animals. Median IL-18 concentration in the WT cohort was 177.4 pg/mL, but we were unable to detect IL-18 in the plasma of IL-18BPKO mice (Figure 6A). This surprising finding could be explained by uncontrolled IL-18 uptake, reduced IL-18 production, and/or compromised IL-18 release among IL-18BPKO mice. The source(s) of circulating steady-state IL-18 have not been established. However, since we had already observed splenic NK cell differences, we targeted the spleen for our inquiry into IL-18 production and expression. Being home to DCs, resident macrophage, and a ready pool of monocytes, we believed we would be able to detect IL-18 perturbations, if they were apparent.

We first examined IL-18 distribution among splenic sections from WT (*N* = 10) and IL-18BPKO (*N* = 10) mice. Antibody specificity was determined using IL-18KO tissue (Figure 6B). Histological results revealed similar patterns of IL-18 distribution among splenic tissue (Figure 6B). IL-18 was richly associated with red pulp macrophage, yet also localized to the marginal zone as well as the interior of the white pulp among both IL-18BPKO and WT mice. These results implied that IL-18 is produced and distributed normally in IL-18BPKO mice.

Although our histological data provided evidence that IL-18 is present in equal abundance in tissue, we also examined splenic lysates. It is previously been shown that murine splenic lysates possess detectable IL-18 in the pro-form (48). We similarly observed pro-form IL-18 (~23 kD) in equivalent abundance from IL-18BPKO and WT mice (Figure 6C). We observed no cleaved mature IL-18 among our lysates from either genotype of mouse. These data provided additional evidence that IL-18 production is comparable between WT and IL-18BPKO mice and that it exists chiefly as the pro-form.

### Peritoneal Macrophage from Wild Type and IL-18BPKO Mice Release Comparable Amounts of IL-18 following LPS and Nigericin Stimulation

Release of mature IL-18, while incompletely understood, is thought to require two steps: priming and activation (49, 50). To determine if IL-18BPKO mice were capable of releasing mature IL-18, we cultured thioglycollate-elicited peritoneal macrophage (TEM) in the presence or absence of LPS (priming) and then followed with nigericin (activation). Nigericin, a pore-forming potassium ionophore, alters cellular potassium levels and activates the NLRP3 inflammasome, leading to caspase 1 activation and subsequent IL-18 maturation and release (51, 52).

With this approach, we observed no deficiency in IL-18 release among IL-18BPKO macrophage exposed to increasing concentrations of nigericin (Figure 6D). Importantly, the IL-18 ELISA shows less than 10% cross reactivity with pro-form IL-18 (manufacturer’s communication). Thus, the measured IL-18 levels are not unduly influenced by passive pro-form IL-18 release from necrotic cells. In addition, we found that IL-18BPKO and WT macrophage produced comparable amounts of IL-1β following nigericin treatment, as well as comparable IL-6 amounts following the initial LPS stimulation (Figure S4 in Supplementary Material). From these results, we conclude that IL-18BPKO TEM responded normally to established stimuli. In total, the analysis of splenic tissue and macrophage responses indicate that there is no deficiency in IL-18 production or release in IL-18BPKO mice and that primary macrophage respond relatively similarly between the two genotypes. Therefore, the severe reduction of IL-18 in the plasma may be resulting from uncontrolled IL-18 uptake or some form of IL-18 mismanagement in the absence of the IL-18BP.

### IL-18BPKO Mice Exhibit Normal Weight Gain

**Figure 7:**
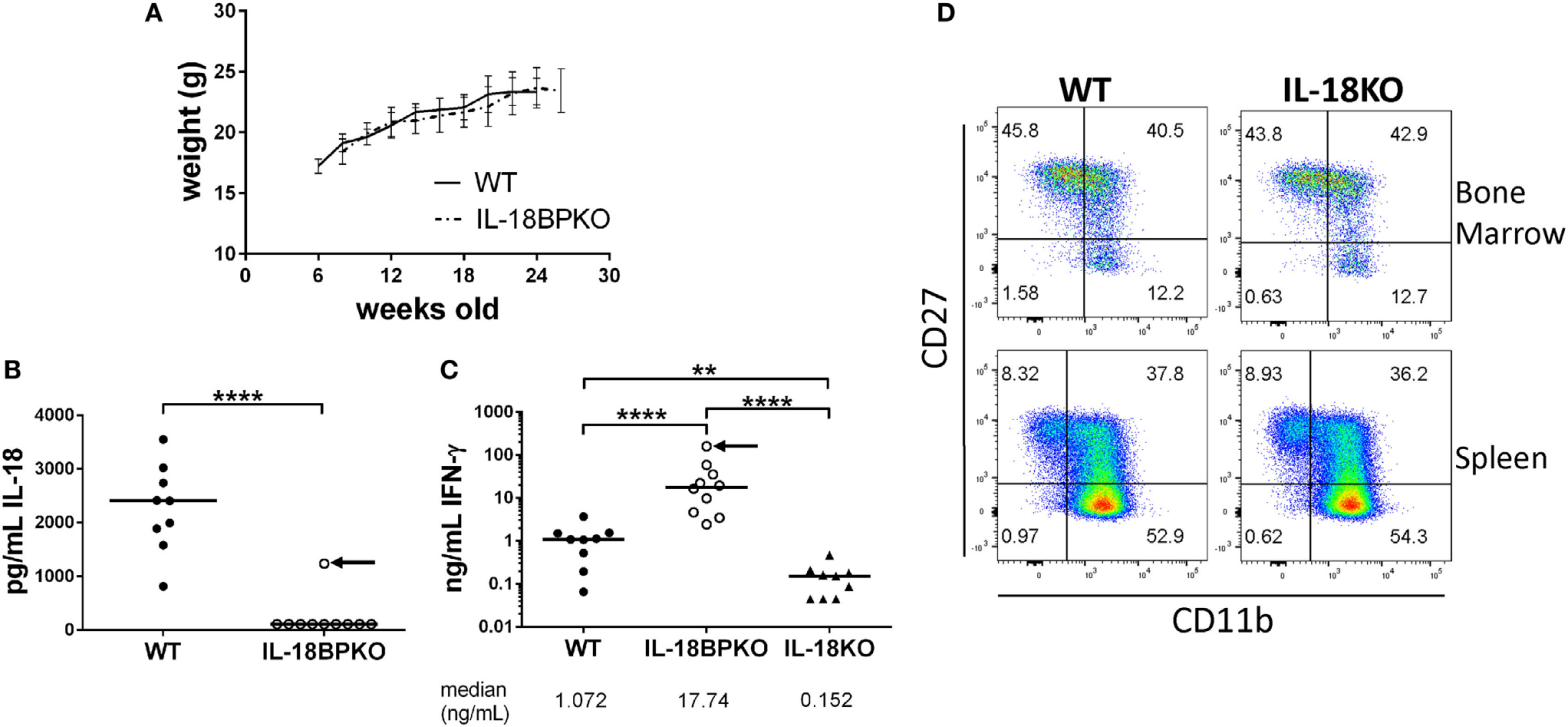
While circulating IL-18 is substantially reduced in interleukin (IL)-18BPKO mice, there is no indication of attenuated IL-18 signaling. **(A)** We followed five wild-type (WT) and six IL-18BPKO female mice for several weeks to observe changes in weight that had been associated with reduced IL-18 signaling. We observed no difference in weight gain between WT and IL-18BPKO mice. Bars represent SD for mean weight measurement. **(B)** Submandibular plasma was isolated 8 h post intraperitoneal LPS (35 µg) injection. In 9/10 IL-18BPKO mice, circulating IL-18 was below limit of detection while WT IL-18 levels increased approximately 10-fold from steady-state levels. **(C)** Plasma IFN-γ levels following LPS challenge were significantly elevated in IL-18BPKO mice compared to WT and IL-18KO mice. Black arrow represents the same IL-18BPKO mouse. **(D)** The absence of IL-18 does not alter proportions nor abundance of natural killer (NK) cell subsets in bone marrow or spleen. Furthermore, total NK cells in IL-18KO mice were comparable to WT (data not shown). Splenic and bone marrow NK cells were identified as in Figure 1 and Figure S2 in Supplementary Material above. Representative results of four separate animals per genotype from two unique experiments are shown. For **(B,C)**, bars represent median. Significance calculated with Mann–Whitney *U* test. **p < 0.01, ****p < 0.0001. For **(C)**, *p* < 0.0001 with Kruskal–Wallis test.

The absence of IL-18 has been associated with obesity in mice that is observable between 20 and 24 weeks of age (53, 54). Since we were unable to observe circulating IL-18 in IL-18BPKO mice, we reasoned that IL-18BPKO mice may be prone to obesity. This could indicate that IL-18 signaling is obstructed without IL-18BP. We followed WT (*N* = 5) and IL-18BPKO (*N* = 6) female mice until at least 24 weeks of age in order to observe any change is weight gain (Figure 7A). While we cannot rule out spontaneous obesity at much later time points, we did not observe differences between WT and IL-18BPKO mice that were comparable to published reports on the absence of IL-18. This suggests that the absence of circulating IL-18 observed with IL-18BP deficiency does not induce metabolic differences similar to those seen in IL-18KO mice, implying functional IL-18 signaling remains intact.

### IL-18BPKO Mice Produce Elevated Levels of Circulating IFN-γ following LPS Stimulation

LPS challenge leads to increased circulating IL-18 as well as increased IL-18-dependent IFN-γ production (34, 37). However, in an inflammatory environment lacking IL-18BP, amplified and uncontrolled IL-18 signaling could result in heightened IFN-γ responses. To test this hypothesis, we administered LPS to WT (*N* = 9), IL-18BPKO (*N* = 10), and IL-18KO (*N* = 9) female mice and recovered plasma 8 h following injection. We then tested for circulating IL-18 and IFN-γ levels. Among WT mice, circulating IL-18 levels were substantially increased from the steady-state conditions shown above and were significantly higher than among IL-18BPKO mice (Figure 7B). Indeed, in 9 out of 10 IL-18BPKO mice, circulating IL-18 was again not detectable. By contrast, we observed grossly elevated IFN-γ levels among IL-18BPKO mice compared to WT (Figure 7C). Interestingly, the IFN-γ level was highest (174.3 ng/mL) from the single IL-18BPKO mouse (denoted by black arrow in Figures 7B,C) in which we detected circulating IL-18 (1235.8 pg/mL). By contrast, IL-18KO mice produced significantly lower IFN-γ than either WT or IL-18BPKO animals following LPS challenge. These data indicate that IFN-γ responses are magnified in the absence of IL-18BP, further indication of functional, yet uncontrolled, IL-18 signaling.

### IL-18KO Mice Show Normal NK Cell Maturation Patterns

Mice lacking IL-18BP have severely reduced quantities of circulating IL-18, as well as aberrant proportions of NK cell subsets. To gauge how the absence of IL-18 may influence NK cell maturation according to the CD27 and CD11b paradigm, we analyzed splenic and bone marrow NK cells from female IL-18KO (*N* = 4) and WT mice (*N* = 4). We observed no difference in proportion or number of NK cells or NK cell subsets among IL-18KO mice (Figure 7D and data not shown). These data demonstrate that IL-18 is not required for normal NK cell maturation. Thus, NK cell differences in IL-18BPKO mice cannot be explained by insufficient or absent IL-18 signaling.

### *In Vitro* IL-18 Stimulation of Splenocytes Is Capable of Modulating Abundance of NK Cell Subsets

The absence of IL-18BP creates skewed abundance and functional differences among NK cells, including an expanded subset of IL-18Rα^−^ NK cells. We also observed a severe reduction in circulating IL-18 among IL-18BP-deficient animals, but we found no defect in IL-18 expression or release. Furthermore, we found that animals lacking IL-18 possess phenotypically normal NK cells. In the absence of IL-18BP, it can be assumed that available IL-18R heterodimers present on the plasma membrane of leukocytes (e.g., neutrophils, basophil, NK cells, T cells, and DCs) would be freely available for unimpeded IL-18 signaling. Such signaling could be responsible for NK cell alterations we observed. IL-18 has been reported to both enhance or be detrimental to NK cell survival (11, 55) and it is currently unknown how IL-18 signaling may impact proportions of NK cell subsets. To gauge if/how IL-18 signaling alters abundance of CD27 and CD11b subsets, we cultured splenocytes from WT and IL-18BPKO mice for 24 h in the presence of IL-18.

**Figure 8:**
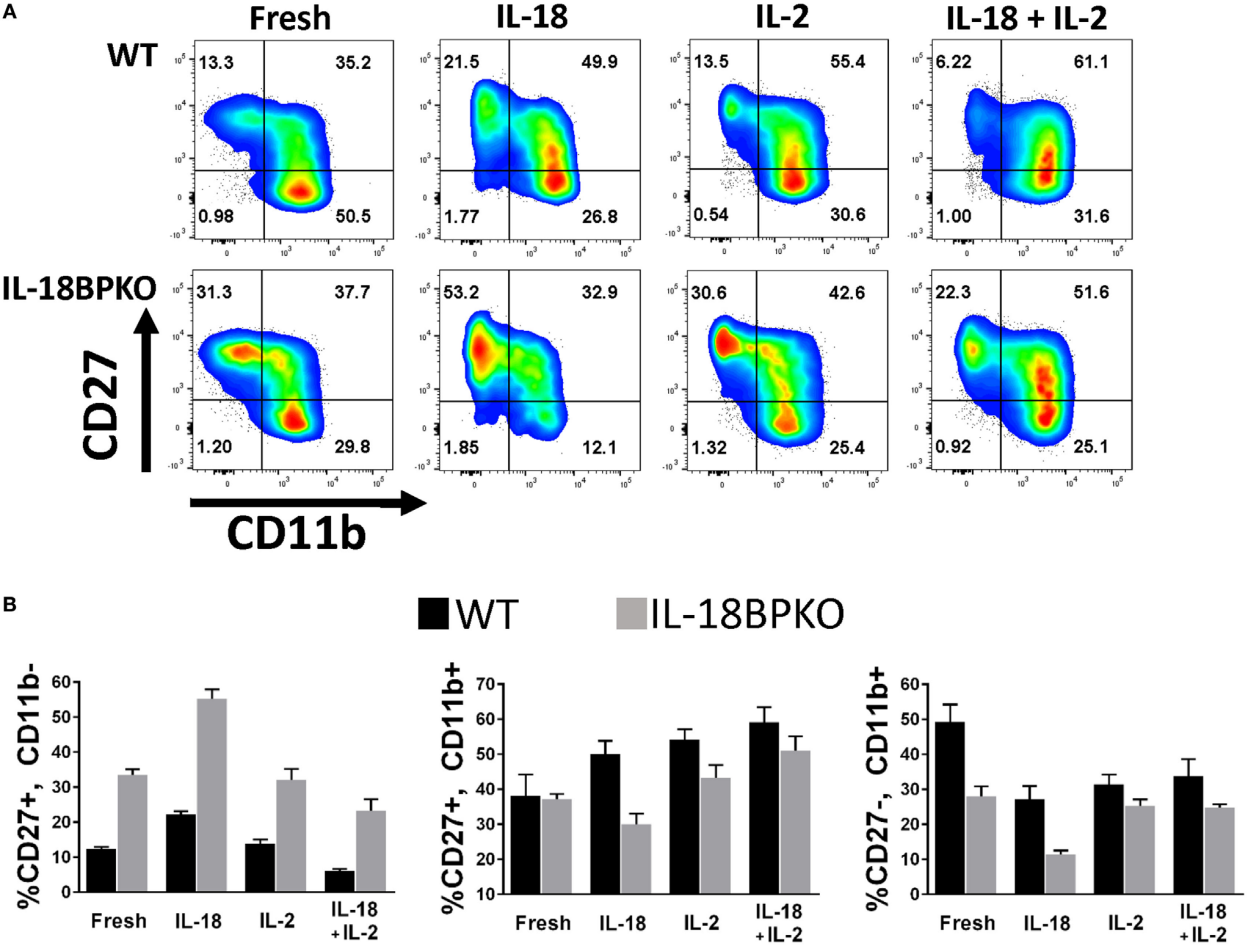
CD27 and CD11b proportions are altered following 24 h culture with interleukin (IL)-18. We cultured splenocytes with IL-18 (50 ng/mL), IL-2 (2 ng/mL) or both IL-18 and IL-2 overnight to observe if IL-18 is capable of altering proportions of CD27 and CD11b-expressing subsets from their proportions immediately following isolation (fresh). **(A)** Representative results from one wild type (WT) and one IL-18BPKO. **(B)** IL-18-treated cultures possessed increased proportions of CD27^+^, CD11b^−^ natural killer (NK) cells and reduced proportion of CD27^−^, CD11b^+^ NK cells, an effect similar to what we observed in IL-18BPKO mice. IL-18 in the presence of IL-2 reduced proportions of CD27^+^, CD11b^−^ NK cells while increasing CD27^+^, CD11b^+^ proportions and either decreasing (WT) or having no effect (IL-18BPKO) on CD27^−^, CD11b^+^ proportions. Results of three separate experiments, one WT, and one IL18BPKO per experiment. Bars denote mean values and error bars represent SD.

The data from the stimulations are shown in Figure 8 with a fold change summary in Table 1. Compared to proportions immediately after isolation (fresh), short-term culture with IL-18 resulted in increased proportions of CD27^+^, CD11b^−^ NK cells with reduced proportions of CD27^−^, CD11b^+^ NK cells in both WT and IL-18BPKO cultures. Interestingly, while proportions of CD27^+^, CD11b^+^ NK cells were elevated in the presence of IL-18 among WT cultures, they were somewhat reduced among IL-18BPKO cultures. From these results, it appears that abundant free IL-18 induces and produces proportional changes similar to what we observed with IL-18BPKO mice, that is, elevated CD27^+^, CD11b^−^ proportions with concomitant reductions in CD27^−^, CD11b^+^ proportions.

**Table 1:** Fold change in proportion of natural killer (NK) cell subsets following interleukin (IL)-18 and IL-2 stimulations compared to fresh NK cells.

	%CD27^+^, CD11b^−^		%CD27^+^, CD11b^+^		%CD27^−^, CD11b^+^	
	Wild type (WT)	IL18BPKO	WT	IL18BPKO	WT	IL18BPKO
IL-18	0.80	0.65	0.31	−0.19	−0.45	−0.59
IL-2	0.13	−0.04	0.42	0.17	−0.36	−0.10
IL-18^+^ IL-2	−0.50	−0.31	0.54	0.38	−0.31	−0.12

*It is a color continuum from green to red. Green represents highest fold increase, while red represent lowest fold decrease*.

Intriguingly, results differed in presence of IL-2. While IL-2 alone did not alter CD27^+^, CD11b^−^ proportions compared to fresh, IL-2 with IL-18 reduced the proportions of CD27^+^, CD11b^−^ NK cells in both WT and IL-18BPKO cultures, opposite to what was observed with IL-18 alone. Among WT cultures, IL-2 stimulation with or without IL-18 resulted in increased CD27^+^, CD11b^+^ and reduced CD27^−^, CD11b^+^ proportions, similar to IL-18 alone. Finally, while IL-18BPKO cultures responded similarly for CD27^+^, CD11b^+^ proportions, the CD27^−^, CD11b^+^ proportions remained relatively unchanged. The combined results indicate that IL-18 as well as IL-2 are capable of shifting proportions of NK cells subsets. Furthermore, there are unique responses to identical stimuli among CD11b^−^ expressing NK cells subsets from WT and IL-18BPKO mice.

### Splenic IL-18BP Production Appears to be Centralized among Cells Expressing Mac-3 and IL-18 That Reside in T Cell Rich Zones

**Figure 9:**
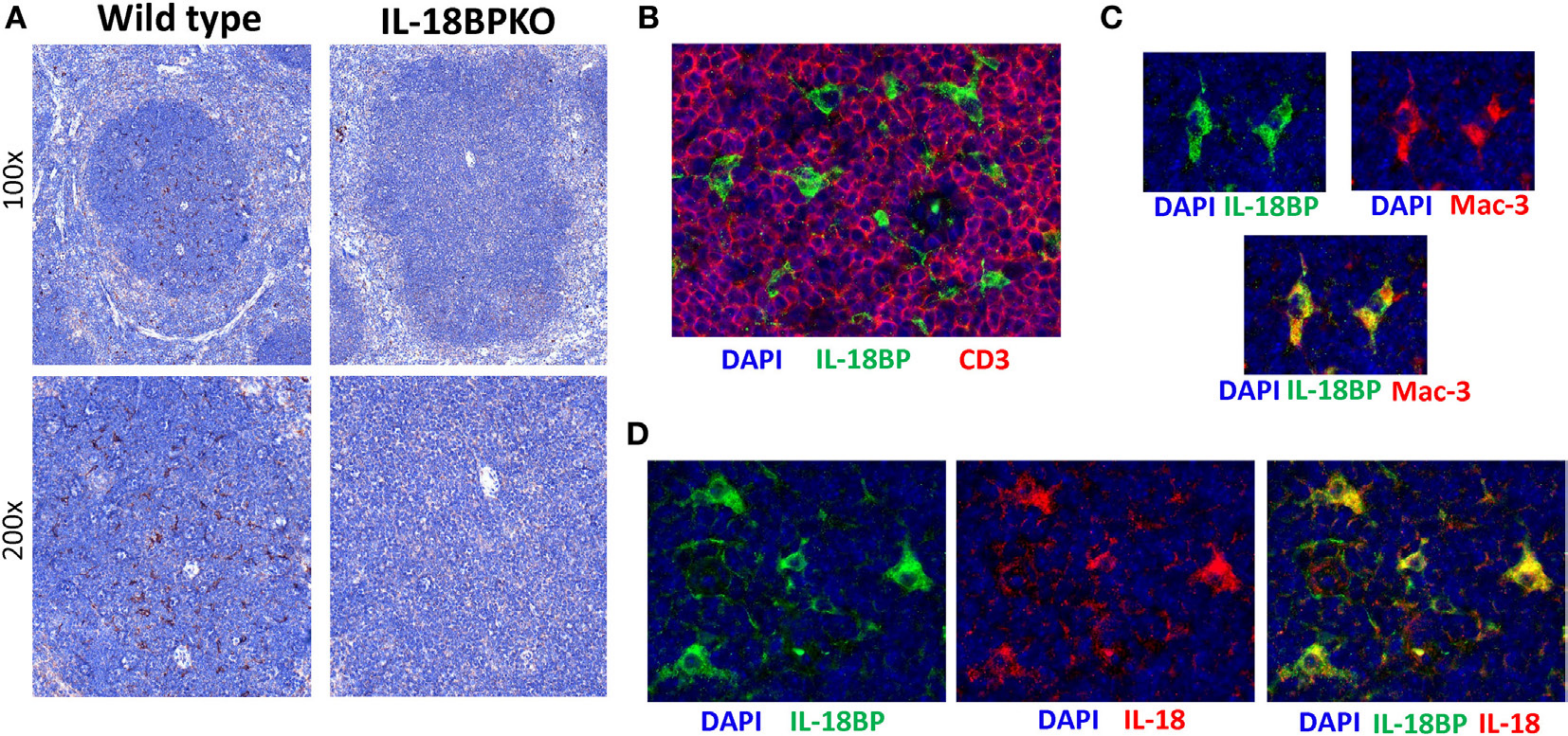
Interleukin-18 binding protein (IL-18BP) expression is localized to the splenic T cell-rich regions among cells expressing both Mac-3 and IL-18. **(A)** Representative IL-18BP immunohistochemistry results from splenic sections from wild-type and IL-18BPKO animals. Unlike the pattern of distribution seen for IL-18, IL-18BP was not prominent among the red pulp. Rather, IL-18BP-expressing cells were relatively abundant in the white pulp and appear to have a dendritic morphology. **(B)** Further examination of IL-18BP using immunofluorescence revealed that IL-18BP-expressing cells (green) reside in the T cell-rich zone of the spleen and are generally surrounded by CD3-expressing cells (red). **(C)** In addition, IL-18BP-expressing cells (green) were found to express Mac-3 (red), a marker expressed by macrophage as well as dendritic cells. **(D)** Finally, IL-18BP-expressing cells (green) also produce IL-18 (red). For **(B–D)**, blue is DAPI and images were taken at 400× magnification. Data are representative of splenic sections from eight mice.

Interleukin-18 binding protein plays a profound role in NK cell development and function. However, the identity and localization of IL-18BP-expressing cells in lymphoid tissue is unclear. IL-18BP transcripts have been reported in the murine spleen (18). Based on these findings and the results from our exploration of IL-18 distribution, we presumed that a similar pattern of distribution would be seen with IL-18BP. To test this, we compared IL-18BP expression between WT and IL-18BPKO mice. Surprisingly, IL-18BP localized to the splenic white pulp among cells having a dendritic morphology (Figure 9A), but generally lacking from the splenic red pulp where we had observed substantial IL-18 expression. These data suggest that IL-18 and IL-18BP are not equivalently expressed among splenocytes and points to unique IL-18 management specialization by a DC or macrophage.

To further investigate splenic IL-18BP *in situ*, we performed IF experiments on wild-type mice to establish localization, lineage, and co-expression of IL-18. As suggested from the IHC data, IL-18BP-expressing splenocytes were generally localized to the T cell rich, CD3-expressing zones of the splenic white pulp (Figure 9B). Furthermore, IL-18BP-expressing cells were also found to express Mac-3 (lysosome-associated membrane glycoprotein 2, CD107b), which denotes a DC or macrophage (Figure 9C). Finally, we observed that IL-18BP-expressing cells in splenic white pulp also expressed IL-18 (Figure 9D). Combined with data above on IL-18 distribution within the spleen (Figure 6B), these results generate further questions as to the source(s) of IL-18BP and IL-18 that appears to be crucial in normal NK cell differentiation within the spleen. Either the IL-18BP-expressing cells identified have the unique role of attenuating IL-18 signal throughout the spleen, or this is governed by an extrinsic cellular source and is conveyed to the spleen *via* the blood stream.

## Discussion

In this study, we have assessed some of the immunological consequences of IL-18BP deficiency. Our results reveal that the IL-18BP plays a key role in NK cell maturation and function. While disrupted IL-18 signaling has been shown to negatively impact NK cell function (33, 34), we demonstrate here that absence of IL-18 has no effect on normal maturational patterns of development based upon CD11b and CD27 expression. Rather, it is only in a system of uncontrolled IL-18 signaling that such NK cell maturation is reshaped.

The spleen houses the largest quantity of NK cells within the murine body and, thus, is a major reservoir for systemic trafficking and as well as recruitment to inflamed tissue (56). Our data indicate that overall abundance of splenic NK cells is reduced in the absence of IL-18BP. Although we cannot rule out a novel, undescribed function for the IL-18BP, this cellular reduction is likely due to unrestrained IL-18 signaling. These results are in line with other reports indicating IL-18 alters leukocyte abundance. For example, subcutaneous administration of IL-18 in BALB/c mice has been found to reduce abundance of circulating white blood cells, including lymphocytes (57). Reduced quantities of NK cells have been associated with increased IL-18 levels in HIV as well as in autoimmunity (55, 58). Furthermore, the results of a phase I study on the effects of IL-18 administration among advanced cancer patients revealed a large contraction of NK cells after IL-18 treatment (59).

Investigations into how IL-18 leads to reduced NK cell numbers suggest key roles for TNF-α and FasL promoting NK cell death (55, 58). Although we did not analyze NK cells for the presence of FasL in our studies, we did examine NK cells for production of TNF-α. Indeed, we observed heightened TNF-α production by NK cells from IL-18BPKO mice. However, unlike IFN-γ, TNF-α production was not augmented with IL-18 stimulation and appears to be directed by an alternate factor or factors. Nevertheless, the increased capacity for TNF-α production by IL-18BPKO NK cells could result in NK cell death, perhaps by a mechanism similar to the TNF-α-mediated fratricidal killing observed among human NK cells following IL-18 stimulation (55). In this scenario, the expansion of immature CD27^+^, CD11b^−^ NK cells in IL-18BPKO mice could be interpreted as a compensatory mechanism for the severe attrition within the CD27^−^, CD11b^+^ compartment.

The identification of an IL-18Rα^−^ NK cell subset increases the complexity of models of NK cell differentiation. We found that IL-18Rα^−^ NK cells are phenotypically similar to IL-18Rα^+^ NK cells, expressing a broad range of NK cell-associated activating and inhibitor surface proteins. This population resided within the CD27^+^, CD11b^−^ compartment, which has been shown to be one of the earliest to repopulate tissues following NK cell lineage ablation and reconstitution (38, 40, 41). Importantly, in our evaluation of NK cells using CD27 and CD11b, we did not find a difference among CD27^−^, CD11b^−^ (DN) NK cells, which have been positioned as the progenitors of CD27 and CD11b-expressing subsets (40). While we did analyze the DN subset for IL-18Rα, we observed mostly detectable expression and found that the bulk of the DN subset in our analysis appeared to be dim CD27 or dim CD11b NK cells (data not shown). Therefore, we are unable to speculate on the relationship between DN NK cells and IL-18Rα^−^ NK cells. By phenotypic measures, the IL-18Rα^−^ NK cell subset appears to consist of *bona fide* immature NK cells lacking IL-18 responsiveness. However, future work to query the cytotoxic potential and progenitor status of this subset must be conducted before such conclusions can be drawn. Regardless of its function, the presence of additional heterogeneity within the NK cell lineage using standard gating strategies should be taken into account in future studies on NK cells.

The regulatory factors that govern the levels of circulating IL-18 are unknown. Importantly, the data presented here suggest that IL-18BP is required for normal levels of circulating IL-18. In this regard, it may be more appropriate to consider plasma IL-18BP as a form of carrier protein, similar in function to plasma steroid-binding proteins (60). In the absence of IL-18BP, soluble IL-18R (61, 62) and IL-18R-bearing cells (such as NK cells, T cells, and granulocytes) are freely competitive for available IL-18, which could directly limit its systemic availability and lead to potentially unwanted cellular activation. Alternatively, when IL-18BP is present, its ability to form complexes with IL-18 prevents acquisition by IL-18R-bearing cells, allowing for unimpeded transport throughout the circulatory system. Yet, if IL-18BP does function as a carrier protein, the destination(s) and biological role of IL-18BP:IL-18 complexes are unclear. It is plausible that these complexes are being conveyed to tissues throughout the body to supply IL-18 signaling or that they are destined for degradation and elimination.

Our evaluation of IL-18 and IL-18BP within splenic tissue revealed disparate patterns of distribution for these two proteins. While IL-18 appears to be abundantly expressed within the red pulp and less so in the white, IL-18BP is limited to a smaller subset of Mac3^+^, IL-18^+^ cells generally residing in the T cell rich zones of the white pulp. It is unknown if these IL-18BP-producing cells are in close proximity to NK cells of any subset. Such proximity seems unlikely, however, based on previous findings that demonstrated the majority of splenic NK cells were localized in the red pulp, near CD11b- and CD11c-expressing cells, likely DCs, macrophage, and monocytes [see Ref. (56, 63)]. Assuming that splenic red pulp DCs/macrophage provide IL-18 to NK cells at their immune synapse (64), any necessary attenuation provided by IL-18BP appears to originate from a non-adjacent cellular source. For this to occur, IL-18BP could be released and distributed throughout the tissue (or provided *via* circulation) in sufficient abundance to dampen the impact of IL-18. Unlike other NK cell cytokines (e.g., IL-12, IL-15, and IL-21), IL-18 protein is readily available from myeloid cells without requiring a stimulus to promote its transcription and translation (65–67). Also, mature IL-18 can be released from monocytes with just ATP stimulation (68), and appreciable circulating levels of biologically active IL-18 are present at steady state in both mice and humans (2). Considering these conditions, there is a high likelihood of intense and unfocused IL-18 signaling in the absence of IL-18BP.

With the goal of evaluating the impact of such intense signaling on NK cell proportions, the results from our short-term stimulus with IL-18 with or without IL-2 suggest that IL-18 stimulation itself is capable of modifying the abundance of NK cell subsets. Importantly, we observed that IL-18 stimulation reduced proportions of CD27^−^, CD11b^+^ NK cells and elevated proportions of CD27^+^, CD11b^−^ NK cells compared to freshly isolated phenotypic proportions. While this demonstrates phenotypic changes similar to what we observed in IL-18BPKO mice, it does not reveal the mechanism by which these proportions are altered. Yet, if IL-18 is directly capable of leading to NK cell death (55, 58), it is reasonable to predict that we could see such changes since within the CD27^+^, CD11b^−^ compartment resides a IL-18Rα^−^ subset which may be protected from IL-18-induced cell death. A major limitation with these stimulations is that we cannot rule out the gain or loss of expression of CD27 and/or CD11b with any treatment. Such changes of expression could also impact proportions. Follow-up studies designed to clarify how IL-18 signaling impacts NK cells subsets using pre-sorted subsets will provide a definitive answer to this question.

In conclusion, these findings establish that IL-18BP is required for normal NK cell abundance and function. Its deficiency results in aberrant proportions of NK cell subsets, and NK cells are uniquely polarized to TNF-α production. These cellular changes combined with the reduction of circulating IL-18 in the absence of IL-18BP indicate that IL-18BP operates somewhat like a carrier protein. In translating these insights into the human system for clinical relevance, the scenario becomes slightly more complicated due to the presence of IL-37 in humans. IL-37 is an anti-inflammatory IL-1 family member that signals through IL-18Rα and SIGIRR (69, 70) and has not been identified in mice. Interestingly, a complex of IL-37 and IL-18BP was found to inhibit IL-18-dependent IFN-γ production by approximately 30% more than IL-18BP alone (71). While this indicates that IL-37 may contribute to IL-18 inhibition in humans, the rather constrained experimental conditions required to induce the effect are generally not observed in human plasma, nor have they been reported from other tissue. Thus, the *in vivo* impact of IL-37 on IL-18BP inhibition is unknown. Nevertheless, the modulation of IL-18 signaling is an attractive therapeutic approach, both by harnessing its stimulating potential (23, 59) as well as by quelling its inflammatory influence (72). Depending on the desired outcome, more than just IL-18 may require manipulation for optimal results.

## Ethics Statement

This study was carried out in accordance with the recommendations of Institutional Animal Care and Use Committee (IACUC) at UNMC. The protocol was approved by the UNMC IACUC committee.

## Author Contributions

RH: experimental design and execution, and writing; AC, KL-A, and KO: experiment execution; and NS: experimental design.

## Conflict of Interest Statement

The authors declare that the research was conducted in the absence of any commercial or financial relationships that could be construed as a potential conflict of interest. The reviewer, SP, and handling editor declared their shared affiliation.
