# Supplementary figures and images for: Interleukin (IL)-18 Binding Protein Deficiency Disrupts Natural Killer Cell Maturation and Diminishes Circulating IL-18
Source: Front Immunol. 2017 Aug 28;8:1020. doi: 10.3389/fimmu.2017.01020 (PMC5581878; doi:10.3389/fimmu.2017.01020)

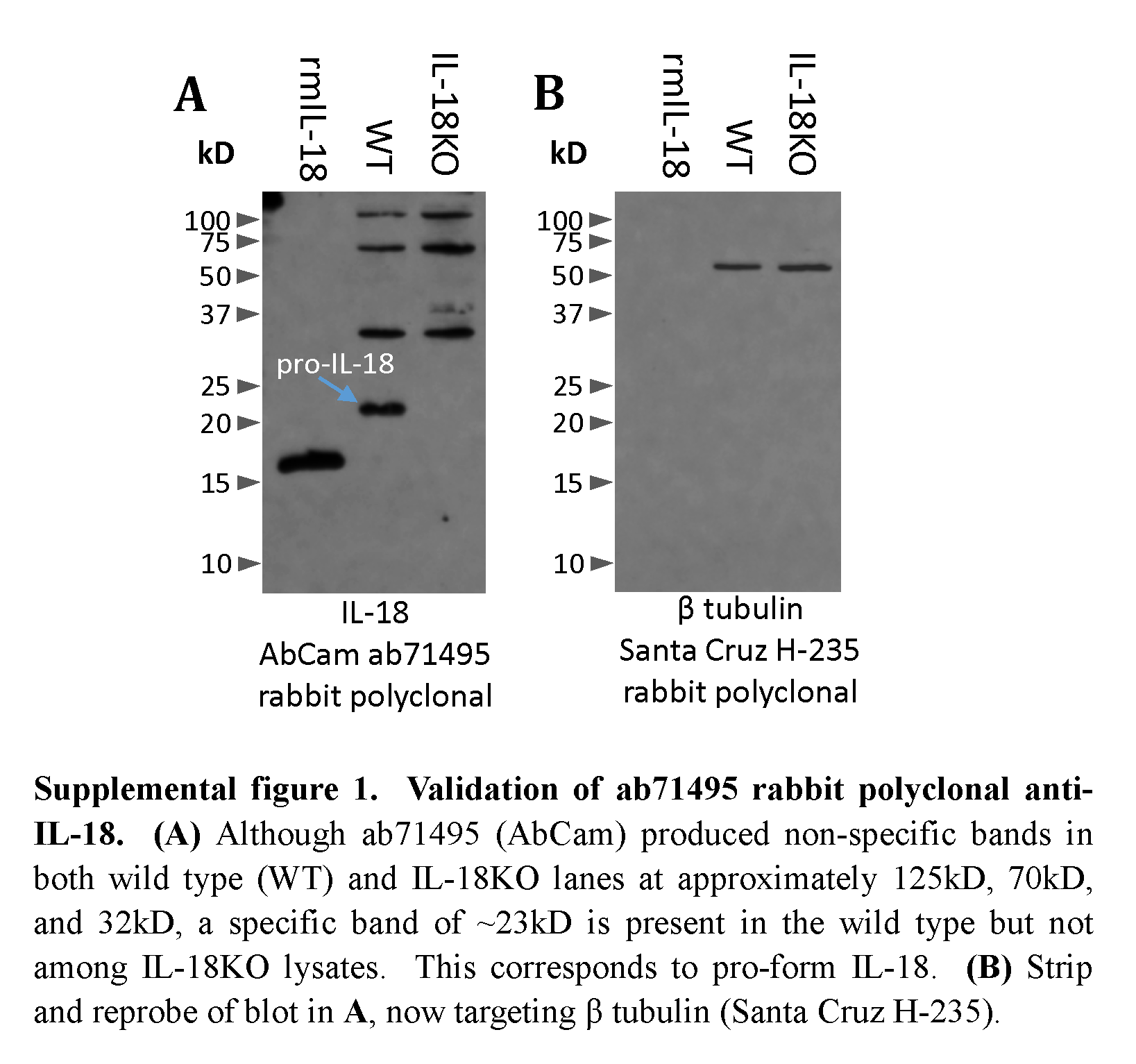

Supplement: Supplementary file 1 [file Image_1.tif]

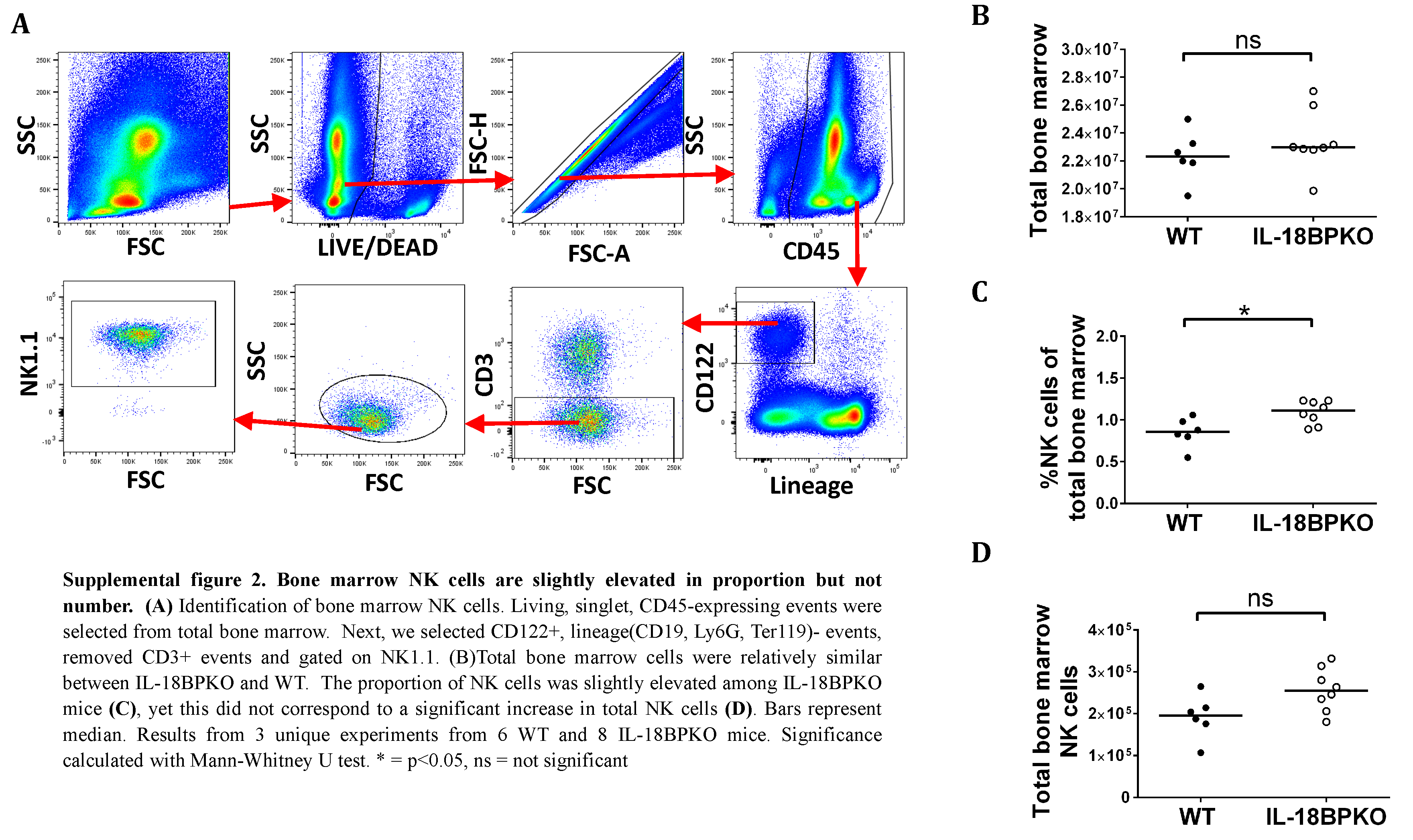

Supplement: Supplementary file 2 [file Image_2.tif]

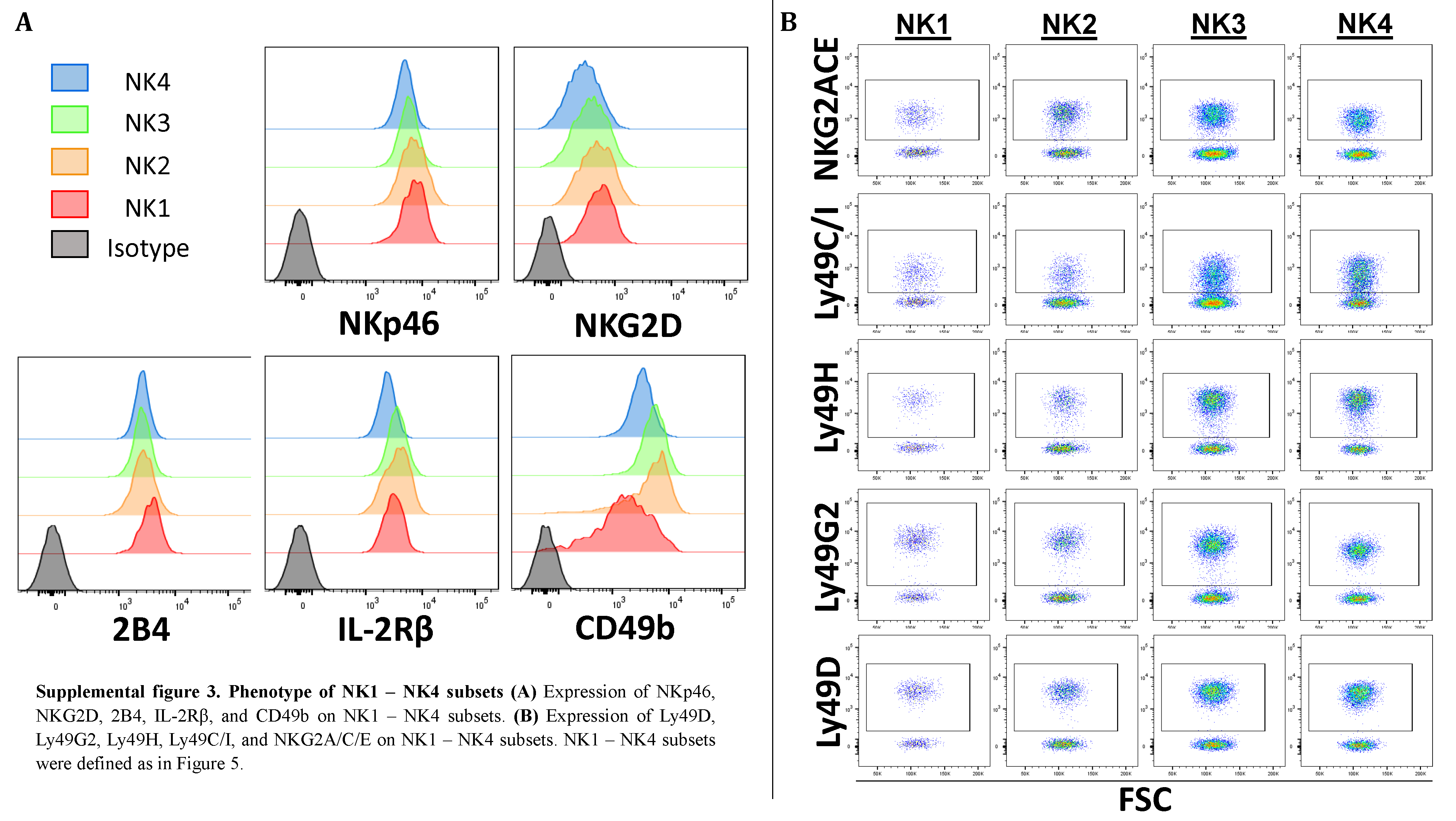

Supplement: Supplementary file 3 [file Image_3.tif]

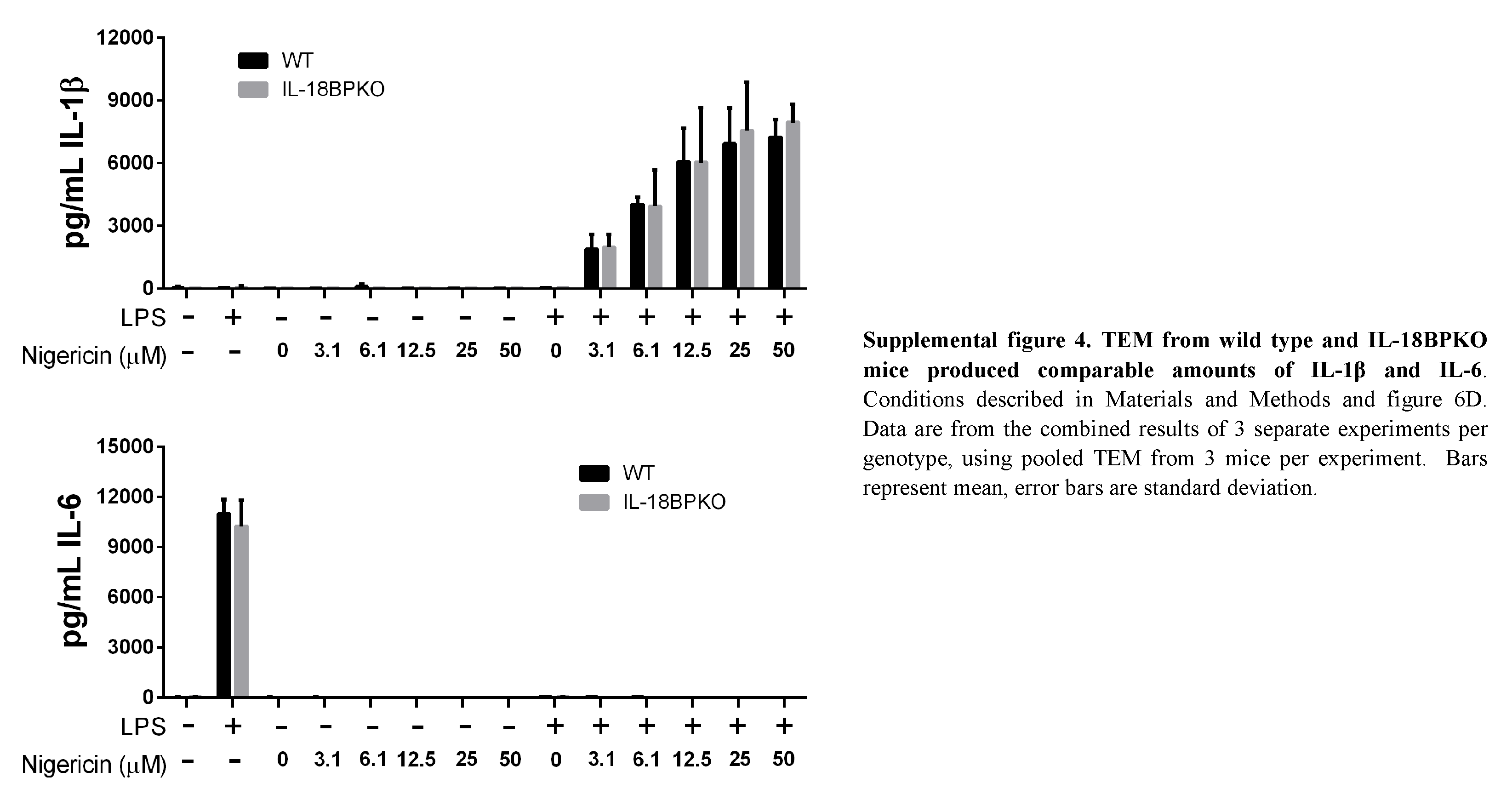

Supplement: Supplementary file 4 [file Image_4.tif]
